# The longevity and reversibility of quiescence in *Schizosaccharomyces pombe* are dependent upon the HIRA histone chaperone

**DOI:** 10.1080/15384101.2023.2249705

**Published:** 2023-08-27

**Authors:** Csenge Gal, Grace A. Cochrane, Brian A. Morgan, Charalampos Rallis, Jürg Bähler, Simon K. Whitehall

**Affiliations:** a Biosciences Institute, Faculty of Medical Sciences, Newcastle University, Newcastle upon Tyne, UK; b School of Biological and Behavioural Sciences, Queen Mary University of London, London, UK; c Department of Genetics, Evolution and Environment and Institute of Healthy Ageing, University College London, London, UK

**Keywords:** HIRA, histone chaperone, quiescence, G0, MBF transcription factor, chromatin

## Abstract

Quiescence (G0) is a reversible non-dividing state that facilitates cellular survival in adverse conditions. Here, we demonstrate that the HIRA histone chaperone complex is required for the reversibility and longevity of nitrogen starvation-induced quiescence in *Schizosaccharomyces pombe*. The HIRA protein, Hip1 is not required for entry into G0 or the induction of autophagy. Although *hip1*Δ cells retain metabolic activity in G0, they rapidly lose the ability to resume proliferation. After a short period in G0 (1 day), *hip1*Δ mutants can resume cell growth in response to the restoration of a nitrogen source but do not efficiently reenter the vegetative cell cycle. This correlates with a failure to induce the expression of MBF transcription factor-dependent genes that are critical for S phase. In addition, *hip1*Δ G0 cells rapidly progress to a senescent state in which they can no longer re-initiate growth following nitrogen source restoration. Analysis of a conditional *hip1* allele is consistent with these findings and indicates that HIRA is required for efficient exit from quiescence and prevents an irreversible cell cycle arrest.

## Introduction

Quiescence (G0) is the reversible suspension of cell division. Many cells in the human body are quiescent and the ability to enter and exit this state appropriately is essential for tissue repair and regeneration [1]. As such, quiescence is not only important for the coordination of growth and development but also for the maintenance of health. For eukaryotic microbes quiescence represents a mechanism that facilitates survival in adverse nutritional conditions and the majority of microbes in the environment are thought to exist in a quiescent state [2].

Despite its importance, many aspects of quiescence remain poorly understood. Yeasts such as *Saccharomyces cerevisiae* and *Schizosaccharomyces pombe* provide useful model systems to dissect pathways that control quiescence. A population of quiescent cells (called Q cells) can be isolated from stationary phase cultures of *S. cerevisiae* that share key features with quiescent mammalian cells [3]. These include the suspension of cell division, cytoplasmic rearrangements, condensed chromosomes, low levels of protein synthesis, and diminished ribosome biogenesis [2]. Quiescence in *S. pombe* can be induced by nitrogen starvation [4]. The removal of a nitrogen source from exponentially growing cells induces two rounds of cell division followed by arrest approximately 6 h later in a pre-replicative state (1C DNA) [5]. At this point, cells can mate and undergo meiosis if sexual partners of the opposite mating type are available. In the absence of a mating partner, cells enter a long-lived quiescent state after about 12 h which is believed to be the transition into G0. These nitrogen-starved G0 cells have distinct characteristics, they are small and round, stress-resistant and have a long chronological lifespan [5].

Unsurprisingly, entry into and exit from quiescence results in major changes to gene expression. Transcription in *S. cerevisiae* Q cells is subject to a global shutoff [6]. Similarly, in *S. pombe* entry into G0 results in a drastic global shrinkage of the transcriptome [7] and reentry into the vegetative cell cycle is accompanied by major transitions in transcript levels [8]. Quiescence also results in extensive changes to chromatin structure [9] and a variety of chromatin regulators are critical for quiescence entry and maintenance [10]. In some cell types quiescence is associated with the *de novo* formation of heterochromatin. Indeed, the survival of quiescent *S*. *pombe* cells requires the establishment of RNAi- and H3K9me-dependent facultative heterochromatin [11,12]. Similarly, the H4K20 dimethyltransferase, Suv4-20h1 controls quiescence in skeletal muscle stem cells by directing the formation of facultative heterochromatin [13].

Maintenance of chromatin structure in non-proliferating cells would be expected to be heavily reliant on DNA replication-independent chromatin regulators. Consistent with this prediction, the replication-independent histone chaperone complex, HIRA is important for the maintenance of the chromatin landscape in senescent fibroblasts [14]. Furthermore, the analysis of *S. pombe* has suggested a role for HIRA in quiescent cells [15,16]. In humans, the HIRA (or HIR) complex is composed of the HIRA protein and two structurally unrelated proteins called Cabin1 and UBN1 [17,18]. Similarly, in *S. pombe* two HIRA-related proteins (Hip1 and Slm9) form a complex with Hip3 and Hip4, which are homologs of human Cabin1 and UBN1, respectively, [19,20]. HIRA functions in concert with another conserved histone H3-H4 chaperone, Asf1 to mediate the assembly of nucleosomes independently of DNA replication [21]. Consistent with this finding, HIRA is associated with the replication-independent histone variant H3.3 in mammalian cells [22]. Here we demonstrate that the *S. pombe* HIRA complex is critical for the longevity and reversibility of quiescence. HIRA is required for efficient exit from short-term G0 and the induction of genes that are critical for S phase. In addition, HIRA prevents the rapid progression of G0 cells to a permanently arrested senescent state.

## Materials and methods

### Strains and growth media

Genotypes of strains are shown in Table S1. Culture was performed in rich (YES) medium or Edinburgh minimal medium (EMM). To induce quiescence, prototrophic cells were grown in EMM to exponential growth phase, harvested by centrifugation (1000 × g for 2 min), washed 2–3 times in EMM lacking NH_4_Cl (EMM-N), resuspended in EMM-N at an OD_595_ 0.15–0.25 and incubated at 30°C for the required time. Restoration of a nitrogen source was achieved by resuspending cells in YES or plating cells on YES agar.

A strain expressing Hip1 fused to the ER HBD (*hip1-HBD*) was constructed as follows. A DNA fragment was PCR-amplified from ERHBD-kanMX6 [23] using primers HBDXhoI (5ʹ-GCATAGCTCGAGGATCCCCGGGTTCTGCTGGAGACATGAGAGCT-3ʹ) and HBDPciI (5ʹ-GACCTAACATGTTCAGACTGTGGCAGGGAAACC-3ʹ). The fragment was digested with XhoI and PciI and cloned into pRip42-Hip1-CTAP that had been digested with XhoI and NcoI. The resulting plasmid (pRip42-Hip1-HBD) was digested with Bst98I and transformed into wild type cells.

### Flow cytometry

Approximately 10^7^ cells were harvested and then resuspended in 1 ml ice-cold 70% ethanol. A 0.3 ml aliquot was transferred into 3 ml of sodium citrate (pH 7.2) in a 15 ml Falcon tube, mixed and centrifuged at 2000 rpm in a bench top centrifuge for 5 min. The pellet was resuspended in 0.5 ml 50 mM sodium citrate (pH 7.2) supplemented with 0.1 mg/ml RNase A and was incubated at 37°C for 2 h. Nuclei were stained by the addition of propidium iodide to a final concentration of 4 µg/ml. Cells were analyzed using a FACS Canto II flow cytometer (BD Biosciences), data was collected using FACSDiva and analyzed using Cyflogic.

### Protein extracts and western blotting

Preparation of whole cell protein extracts and western blotting was performed as previously described [24].

### Phloxine B staining

Approximately 1.5 × 10^7^ cells were collected and Phloxine B was added to the medium to a final concentration of 5 mg/L. Cells were incubated for 2 h at 30°C with shaking and then washed twice in 1× PBS (pH 7.4).

### ATP levels

The BacTiter-Glo system (Promega, Madison, WI, USA) was used to monitor ATP levels in quiescent cells. An aliquot of 100 *μ*l of quiescent cell culture was mixed with 100 *μ*l BacTiter-Glo reagent in a white 96-well microplate. After 15 min, luminescence was detected using a FLUOstar Omega microplate reader. Luminescence values were corrected for background and cell number.

### RNA sequencing and data processing

RNA was extracted from cells starved for nitrogen for 1 day (−N) and 90 min after the restoration of a nitrogen source (+N) as previously described [25]. Three biological replicates were prepared for each strain under each condition. Stranded RNA sequencing libraries were prepared by Novogene using the NEBNext® Ultra™ RNA Library Prep Kit for Illumina®. Libraries were sequenced using Illumina HiSeq2500. Sequencing reads were aligned to the *S. pombe* Ensembl ASM924v2 genome assembly, using STAR in default mode [26] to generate BAM and bedgraph files. The datasets have been submitted to GEO (accession number: GSE129599).

### Differential gene expression analysis

The cDNA model was downloaded from the Ensemble ASM924v2 genome assembly. Transcripts were quantified over the model using Kallisto [27], in the single end, stranded mode. DESeq2 [28] was used to find differentially expressed genes (Table S2). We used LFC > 1 and FDR < 0.01 to call genes upregulated and LFC <-1 and FDR < 0.01 to call genes downregulated. Gene ontology analyses were performed using ShinyGO 0.77 (http://bioinformatics.sdstate.edu/go/) and AnGeLi (http://bahlerweb.cs.ucl.ac.uk/cgi-bin/GLA/GLA_input)

### RT-qPCR

RNA, extracted as described above, was DNaseI treated either using the Ambion TURBO DNA-free™ Kit or using the Primerdesign Precision DNase kit (DNASE-50). Reverse transcription and quantitative PCR were carried out using Primerdesign Precision OneStep qRT-PCR according to the manufacturer’s instructions. SYBR green detection was recorded using a Rotor Gene 6000 Real-Time PCR machine.

### Micrococcal nuclease (MNase) digestion of chromatin

MNase digestion was performed as described previously [24] except that cell wall digestion was achieved by incubation in 500 µl of CES buffer (50 mM citric acid/50 mM Na_2_HPO_4_ [pH 5.6], 40 mM EDTA [pH 8.0], 1.2 M sorbitol and 10 mM β-mercaptoethanol) containing 7 mg/ml lyticase (Sigma L-5263) and 5 mg/ml lysing enzymes (Sigma L-1412) on a shaker at 30°C for between 2 and 2.5 h

## Results

**Figure 1.**
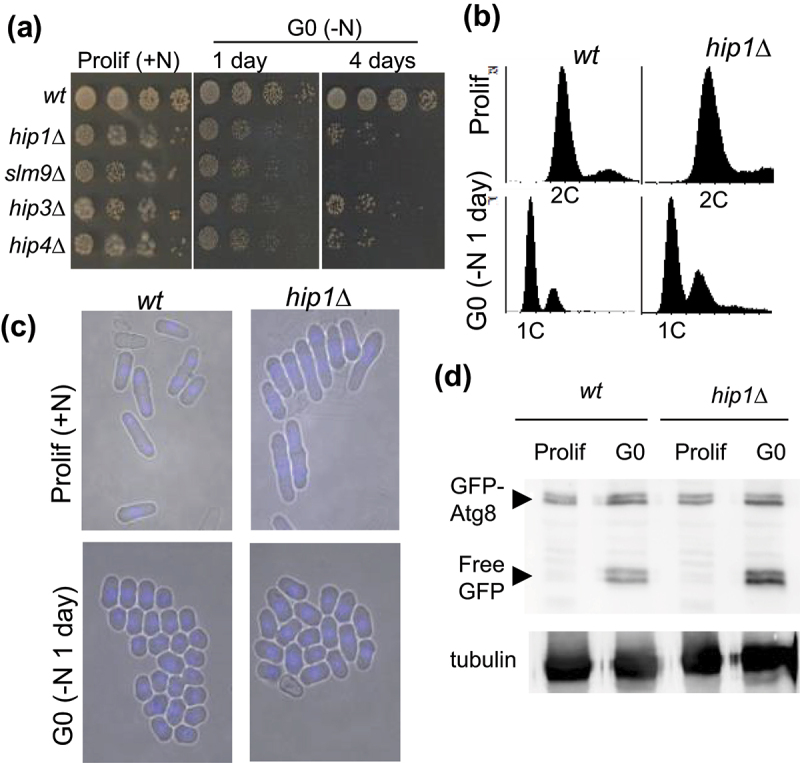
HIRA is required for survival in G0. **(a)** the indicated strains were grown to mid logarithmic phase in EMM medium (Prolif) and then suspended in medium lacking a nitrogen source (EMM-N) at 30°C to induce quiescence (−N). At the indicated time points, an aliquot of the culture was subjected to five-fold serial dilution and then printed onto YES agar plates which were incubated at 30°C for 3–4 days to allow viable cells to form colonies. **(b)** flow cytometric analysis of proliferating cells and cells starved for nitrogen for 1 day. Data are representative of three independent biological repeats. A prominent 1C DNA peak is indicative of a G0 arrest. **(c)** morphology of proliferating cells and cells starved for nitrogen for 1 day (−N). Nitrogen-starved G0 cells adopt a small round morphology. **(d)** HIRA is not required for the induction of autophagy. Wild type and *hip1*Δ cells expressing GFP-Atg8 were grown to mid logarithmic phase in EMM (Prolif) and then resuspended in EMM-N for 1 day (G0). Whole cell protein extracts were prepared and analyzed by western blotting using anti-GFP and anti α-tubulin (TAT-1) antibodies. The presence of free GFP is indicative of the induction of autophagy and α-tubulin levels serve as a loading control. Data are representative of two biological repeats.

Accumulated evidence indicates that deletion of any of the four genes encoding HIRA subunits (*hip1*^+^ *slm9*^+^, *hip3*^+^ or *hip4*^+^) effectively abolishes the function of this histone chaperone complex [15,19,20,29,30]. Therefore, to determine whether HIRA is required for survival in quiescence (G0), we compared the viability of wild type and HIRA mutant strains following nitrogen source depletion. As a defining feature of quiescent cells is the capacity to resume proliferation, viability was initially evaluated by assaying the ability of G0 cells to form colonies when transferred to rich (YES) agar plates. As expected, the majority of wild type cells were viable 4 days after nitrogen removal. In contrast, strains with deletions in genes encoding HIRA subunits (*hip1*Δ, *slm9*Δ, *hip3*Δ and *hip4*Δ), exhibited an extremely severe reduction in survival in G0 that was evident as early as 1 day after nitrogen source depletion (Figure 1a). Given that wild type cells remain viable for months, the impact of loss of HIRA activity was striking.

Previous analysis has indicated that loss of HIRA (*hip1*Δ or *slm9*Δ) does not prevent G0 arrest in response to nitrogen starvation [15,16] and this was confirmed by FACS analysis. Following the removal of nitrogen, the majority of both wild type and *hip1*Δ cells arrested in a pre-replicative (1C) state which was maintained for at least 6 days (Figure 1b and Fig S1). Furthermore, microscopic examination revealed that like wild type cells, *hip1*Δ cells decreased in volume and exhibited a rounded morphology (Figure 1c).

The induction of autophagy is important for viability in quiescence [31,32] and so we monitored autophagy by measuring the cleavage of a GFP-Atg8 fusion protein [33]. Similar levels of free GFP were found in wild type and *hip1*Δ cells 1 day after nitrogen starvation (G0), indicating that HIRA is not required for the induction of autophagy (Figure 1d). Overall, these results suggest that the quiescence defects associated with impaired HIRA function do not result from failure to arrest in G0.

### Loss of HIRA results in a rapid and progressive loss in the ability to resume proliferation

**Figure 2.**
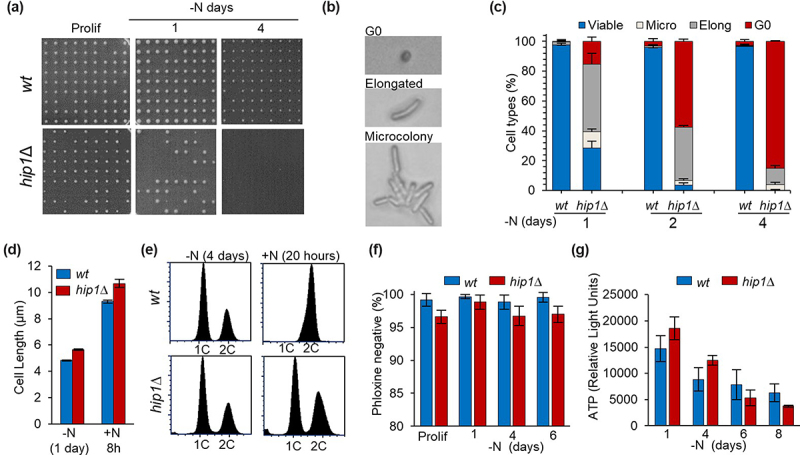
Loss of HIRA results in a progressive loss of capacity to reenter the cell cycle. **(a)** wild type and *hip1*Δ cells were grown to mid logarithmic phase in EMM medium (Prolif) and then resuspended in EMM-N medium (−N) to induce quiescence. At the indicated time points, cell viability was measured by determining the ability of individual cells to form a colony. 81 cells were transferred to defined positions on a YES agar plate using a micromanipulator (Singer). Pates were then incubated at 30°C for 3–4 days to allow cells to form visible colonies. A representative example of the YES agar plates from one of three biological repeats is shown. **(b)** cells treated as described in (A) that failed to form colonies when transferred to a YES agar plate were designated as “inviable”. These cells were further classified based on their morphology using microscopy. Inviable cells that remained as small and round cells were designated “G0”, enlarged but undivided cells as “elongated” and cells that had divided at least once as “microcolonies”. Representative images of the different inviable cell types are shown. **(c)** Percentages of viable and inviable cell types after the indicated times in G0 (−N) were determined as described in (A) and (B). Data are based upon three biological repeats. Error bars are +SEM. **(d)** mean cell length (>100 cells) was determined after nitrogen source depletion for 1 day (−N) and following resuspension in rich (YES) medium for 8 h (+N 8 h). Data are the mean of three biological repeats. Error bars represent ± SEM. **(e)** flow cytometric analysis of G0 cells starved for nitrogen for 4 days (−N 4 days) followed by incubation in fresh YES medium for 20 h at 30°C (+N 20 h). **(f)** the majority of *hip1*Δ cells retain metabolic activity in G0. Wild type and *hip1*Δ cells were grown to mid logarithmic phase in EMM medium (Prolif) and then resuspended in EMM-N medium (−N) and incubated at 30°C for 6 days. At the indicated times cells were stained with Phloxine which is taken up passively but actively exported. As a result, metabolically active “live” cells are non-staining (Phloxine negative). Mean values of Phloxine negative cells from three biological repeats is shown and error bars represent ± SD. **(g)** ATP levels in nitrogen-starved wild type and *hip1*Δ G0 cells were determined using the BactTiter Glo cell viability assay (Promega). Luminescence values were corrected for background and cell number. Mean values were calculated from three biological replicates with each sample assayed in duplicate. Error bars indicate ± SEM.

To provide a quantitative measure of proliferative potential as a function of time in G0, the ability of individual cells to form colonies when re-seeded onto rich agar (YES) plates was determined (Figure 2a–c). Over 90% of wild type G0 cells were capable of forming visible colonies 4 days post nitrogen depletion. By comparison, only ~ 30% of *hip1*Δ cells formed colonies after 1 day of nitrogen starvation, a percentage which declined to ~ 4% by 2 days. Furthermore, after 4 days, less than 1% of the *hip1*Δ cells tested formed a visible colony confirming that impairment of HIRA results in an extremely rapid and progressive reduction in the capacity to resume proliferation.

Cells that failed to form visible colonies were subjected to microscopic examination and classified based on their morphology (Figure 2b,c). After 1 day of nitrogen starvation, only 15% of *hip1*Δ cells remained as small round (G0) cells when re-seeded onto rich (YES) agar. Indeed, the majority of cells that did not form a visible colony had either elongated or undergone a limited number of divisions (designated microcolonies) (Figure 2b,c). Therefore, after short-term G0, most *hip1*Δ cells were capable of re-initiating growth. This was confirmed by measuring the lengths of wild type and *hip1*Δ cells following nitrogen source restoration (Figure 2d and Fig S2). However, after 4 days of nitrogen starvation, the majority (~85%) of *hip1*Δ cells remained small and round when reseeded onto rich (YES) agar (for an example see Figure 2b). Furthermore, FACS analysis confirmed that they were unable to resume proliferation after the restoration of a nitrogen source (Figure 2e).

G0 cells lacking HIRA rapidly progress to a state in which they are incapable of resuming growth and cell division. Therefore, we used staining with the fluorescent dye, Phloxine to determine whether these cells are “alive” or “dead”. Phloxine is taken up passively but is exported by metabolically active (live) cells and so metabolically inactive (dead) cells stain red when treated with this dye [34]. These experiments revealed that even after 6 days in G0 the majority (>90%) of *hip1*Δ cells were metabolically active and were not simply dead (Figure 2f and Fig S3). To confirm this finding we used the BactTiter Glo assay (Promega) to measure ATP levels in wild type and *hip1*Δ G0 cells because metabolic activity is closely related to intracellular ATP content and dead cells do not give a signal in these assays [34]. We found that extended periods in G0 resulted in an overall decline in intracellular ATP, but importantly there was no significant difference in the ATP content of *hip1*Δ and wild type cells throughout the course of the experiment (Figure 2g). Therefore, nitrogen-starved G0 *hip1*Δ cells rapidly lose the ability to re-enter the cell division cycle but they retain metabolic activity and so impairing the function of the HIRA complex results in the premature onset of a senescent state.

**Figure 3.**
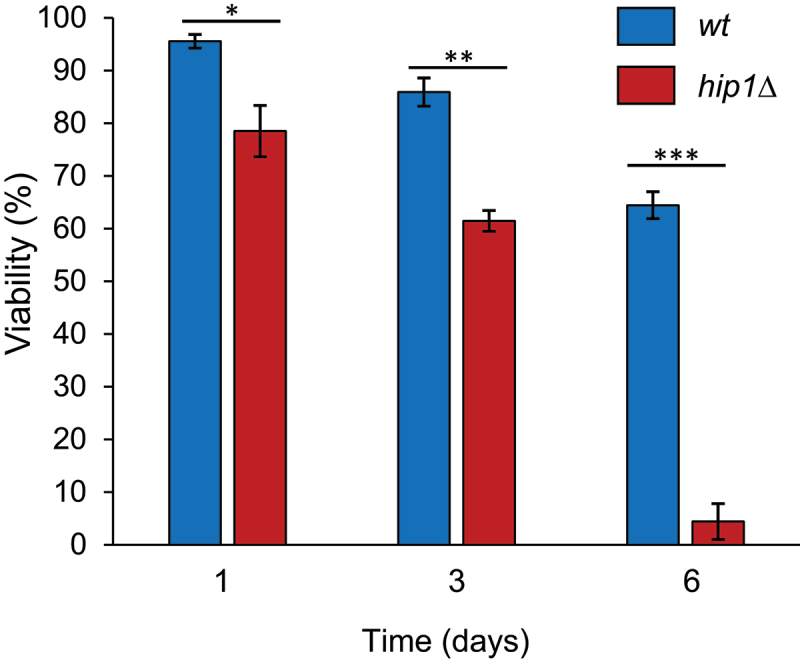
Loss of HIRA reduces survival in stationary phase. Cells from freshly grown YES agar plates were used to inoculate YES medium (50 mL). The resulting YES cultures (*wt* and *hip1*Δ) were then incubated at 30°C with shaking. At the indicated time points after inoculation (1, 3 and 6 days), percentage cell viability was determined by transferring individual cells to defined positions on a YES agar plate using a micromanipulator (Singer). Plates were then incubated at 30°C for 3–4 days to allow colonies to form. Percentage cell viability was calculated from the proportion of cells able to form a visible colony. Mean percentage viability was calculated from three biological repeats. Error bars represent ±SEM. (*p < 0.05, **p < 0.01, ***p < 0.001; t-test).

*S. pombe* cells grown in culture to high density (>2 × 10^8^ cells/ml) stop dividing and enter stationary phase [4]. Unlike nitrogen-starved quiescent cells, the majority of stationary phase cells exit the cell cycle with a post-replicative (2C) DNA content and do not adopt a small rounded morphology. As such, stationary phase represents a distinct non-dividing state [4]. Comparison of wild type and *hip1*Δ stationary phase cultures showed that HIRA is important for viability in this alternative G0 state (Figure 3).

**Figure 4.**
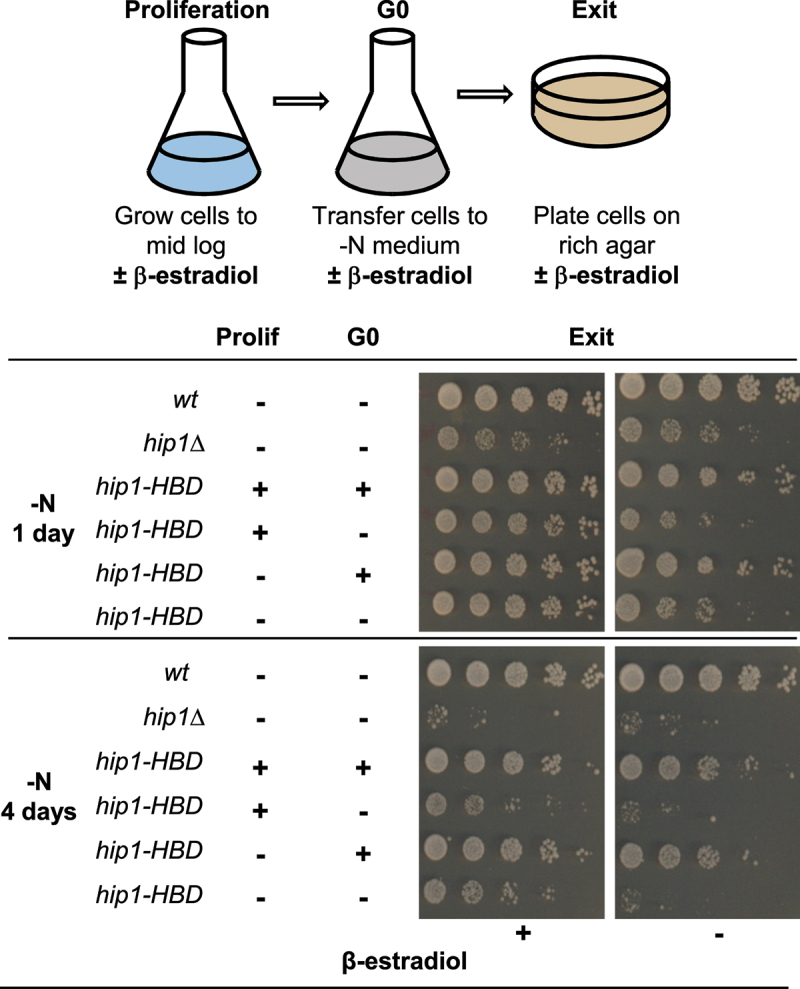
HIRA is required for a reversible cell cycle arrest in G0. The *hip1-HBD* strain expresses Hip1 as a fusion with the hormone binding domain (HBD) of the estrogen receptor [23] which renders Hip1 (and thus HIRA) function dependent upon the presence of β-estradiol in the medium. The top panel shows the experimental scheme. Strains were grown to mid logarithmic phase in EMM (Prolif) and then resuspended in EMM-N at 30°C to induce quiescence (G0). At the indicated times cultures were subjected to five-fold serial dilution and pinned onto YES plates which were then incubated at 30°C for 3–4 days to allow cell proliferation to resume (exit). The absence (-) or presence (+) of β-estradiol (200 nM) in the medium at each stage in the experiment is indicated. Wild type and *hip1*Δ strains were included as controls.

To further investigate the role of HIRA at different stages of G0, a strain (*hip1-HBD*) was constructed in which the HIRA protein Hip1 is expressed as a fusion with the hormone-binding domain (HBD) of the estrogen receptor [23]. In the absence of β-estradiol the HBD tag is sequestered by Hsp90, while the addition of estradiol to the medium releases HBD from Hsp90 thus leading to a rapid induction of function [23]. Proliferating *hip1-HBD* cells were nitrogen-starved to induce entry into G0, incubated for up to 4 days and then transferred onto rich (YES) agar plates to allow exit from quiescence. At each stage in the experiment, the presence of β-estradiol in the medium was manipulated to control whether Hip1 (and thus HIRA), was functional (Figure 4). The presence of β-estradiol at all stages resulted in *hip1-HBD* behaving like wild type, whereas the absence of β-estradiol throughout the experiment resulted in a similar phenotype to *hip1*Δ indicating that the HBD tag provides an effective means of regulating Hip1 function (Figure 4). Importantly, when β-estradiol was present during G0 *hip1-HBD* cells had similar viability to wild type irrespective of whether β-estradiol was present before that stage. Furthermore, the addition of β-estradiol specifically during quiescence exit partially rescued the viability of *hip1-HBD* even after 4 days in G0 (Figure 4). Overall, these results are consistent with the notion that HIRA is dispensable for entry into quiescence but is required for cells to maintain a reversible arrested state.

### Chromatin in quiescent cells lacking HIRA

**Figure 5.**
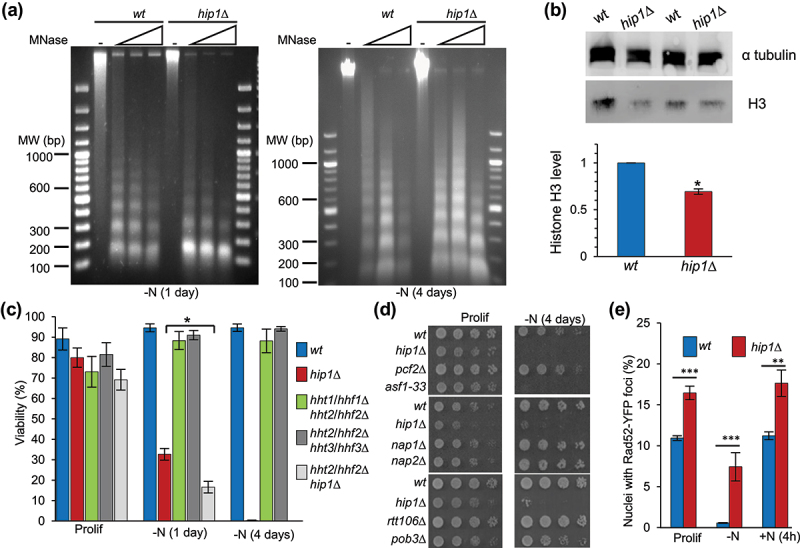
Chromatin and DNA damage in quiescent cells. **(a)** nitrogen-starved G0 cells (1 and 4 days –N) were treated with MNase to digest chromatin and the resulting DNA samples were analyzed on 1.5% TAE agarose gels. Data are representative of three biological replicates. **(b)** G0 cells lacking HIRA have reduced levels of total histone H3. Whole cell protein extracts, were prepared from wild type and *hip1*Δ G0 cells that had been starved for nitrogen for one day. Protein extracts were analyzed by western blotting using anti-histone H3 (Abcam) and anti-α-tubulin (TAT-1) antibodies. Examples of the primary data are shown (above) and a quantification of histone H3 levels normalized to α-tubulin and scaled relative to wild type (below). Data are the mean of four independent repeats. Error bars represent ±SEM (* *p* < 0.05; t-test). **(c)** the indicated strains were grown to mid logarithmic phase in EMM medium (Prolif) and then suspended in EMM-N medium at 30°C (−N) to induce quiescence. At the indicated time points, percentage viability was assayed by determining the ability of cells to form a colony (as described for Figure 2a-c). Data are the mean of at least three biological replicates and error bars represent ± SEM (* *p* < 0.05; t-test). Data for the percentage of viable cells after 1 and 4 days of nitrogen starvation for the wild type and *hip1*Δ strains is the same as that presented in fig 2C and is included for comparison. **(d)** the indicated strains carrying mutations in genes encoding histone chaperones were grown to mid logarithmic phase in EMM medium (Prolif) and then suspended in EMM-N medium for 4 days (−N 4 days). At the indicated times, cultures were subjected to five-fold serial dilution, printed onto YES agar and incubated at 30°C for 3–4 days to allow colonies to form. **(e)** DNA double strand breaks (DSBs) in quiescence and cell cycle reentry. Proliferating (Prolif) wild type and *hip1*Δ cells expressing Rad52-YFP were starved for nitrogen for 1 day (−N). Cells were then suspended in fresh YES medium and incubated for 4 h at 30°C (+N 4hrs). Fluorescence microscopy was used to determine the percentage of nuclei with Rad52-YFP foci which are markers of DSBs. At least 200 nuclei were counted for each strain at each point. Data are the mean of three biological repeats. Error bars represent ± SEM (** *p* < 0.01, *** *p* < 0.001; t-test).

As HIRA mediates replication-independent nucleosome assembly, we hypothesized that deletion of *hip1*^*+*^ may lead to the progressive loss of nucleosomes in quiescent cells resulting in a widespread impairment to chromatin. To investigate this, the global integrity of chromatin was probed using MNase digestion. After 1 day in quiescence, both wild type and *hip1*Δ samples showed clear nucleosomal ladders, a pattern that was maintained even after 4 days in G0 (Figure 5a). Therefore, the loss of proliferative capacity in *hip1*Δ cells does not result from a global loss of chromatin integrity. This does not rule out the possibility that nucleosomes are lost from specific loci in the absence of HIRA, as is the case in proliferating cells [35]. Indeed, quiescent *hip1*Δ cells were found to have lower histone H3 levels relative to the wild type, implying that total nucleosome numbers are reduced in the absence of functional HIRA (Figure 5b and Fig S4).

To investigate if a reduction in histone levels is responsible for the quiescence defects of HIRA-deficient cells we analyzed the viability of strains with deletions in histone genes. *S. pombe* has three histone H3-H4 gene pairs and deletion of the *hht2*^*+*^*-hhf2*^*+*^ gene pair further reduced the viability of quiescent *hip1*Δ cells (Figure 5c). However, single (*hht1/hhf1*Δ, *hht2/hhf2*Δ, *hht3/hhf3*Δ) or double (*hht1/hhf1*Δ *hht2/hhf2*Δ, *hht2/hhf2*Δ *hht3/hhf3*Δ) histone gene pair deletions in a wild type background did not result in any loss of proliferative capacity after 4 days in G0 (Fig S5 and Figure 5c). Thus, reducing histone gene dosage does not phenocopy the G0 defect of *hip1*Δ cells.

To determine whether rapid loss of proliferative capacity in G0 is a common phenotype of histone chaperone mutants we analyzed *nap1*Δ, *nap2*Δ, *pcf2*Δ (CAF-1), *rtt106*Δ, *pob3*Δ (FACT) and *asf1–33* strains (Figure 5d). The *asf1–33* strain [36] had impaired survival in G0 similar to that observed for HIRA mutants (Figure 5d) which is consistent with Asf1 functioning in concert with the HIRA complex [21,30]. However, none of the other histone chaperone mutants showed any detectable loss of proliferative capacity after 4 days in G0. Therefore, HIRA/Asf1 were the only histone chaperones that were essential for survival in short-term quiescence.

### DNA damage in quiescence

Mechanisms that protect against DNA damage are important for maintaining the viability of quiescent *S. pombe* cells. Accumulation of unrepaired damage in G0 manifests as highly elevated levels of dsDNA breaks (DSBs) during the first round of replication during cell cycle reentry [37]. Since HIRA is important for protection against genotoxic stress [29,30], the incidence of DSBs was determined by monitoring the percentage of nuclei with Rad52-YFP foci [38]. In proliferating cells, lack of HIRA (*hip1*Δ) resulted in a small, but significant, increase in spontaneous Rad52-YFP foci (Figure 5e and Fig S6). Rad52-YFP foci were essentially absent in wild type G0 cells, which is consistent with their assembly being a result of lesions generated during DNA replication. Surprisingly, ~8% of the quiescent *hip1*Δ cells were found to contain Rad52-YFP foci suggesting that these cells enter G0 with unrepaired DSBs. Following the restoration of a nitrogen source, increased DSBs were observed in *hip1*Δ cells relative to wild type cells; however, the level was not higher than that observed before G0 arrest (Figure 5e). Therefore, it is unlikely that the loss of viability in G0 that is associated with HIRA-deficient cells results from the rapid accumulation of unrepaired DNA damage.

### Influence of HIRA on the transcriptome of quiescent cells

**Figure 6.**
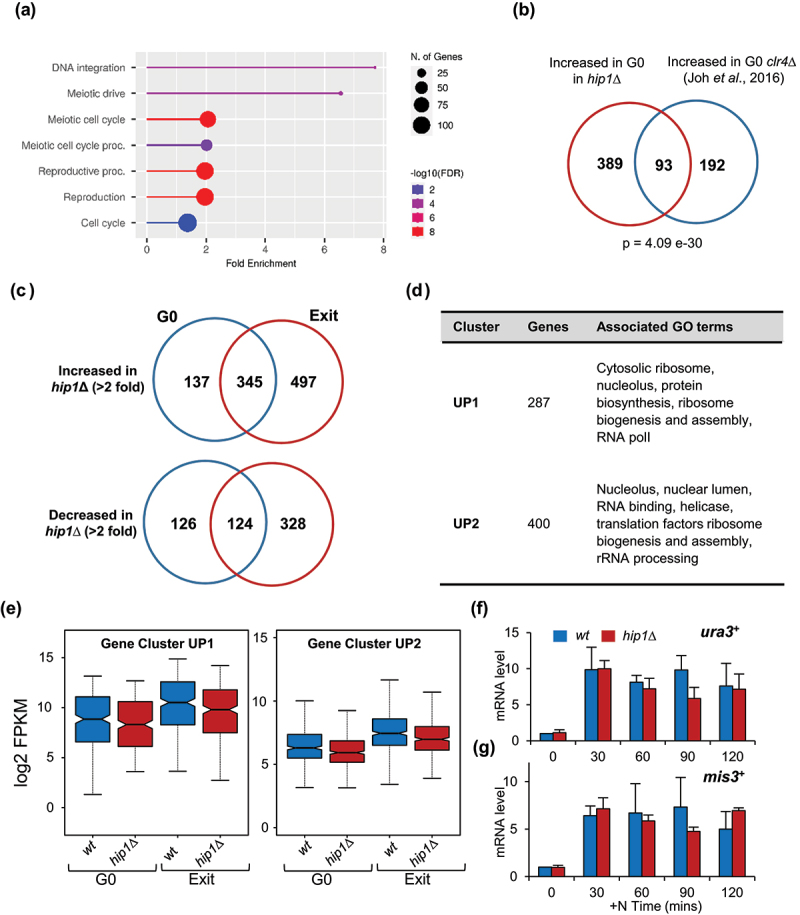
Impact of HIRA on gene expression during quiescence and reentry into the cell cycle. **(a)** Summary of GO biological process terms associated with genes with increased expression (log2 fold change > 1) in G0 *hip1*Δ cells (−N 1 day). FDR was calculated based on a *p* value from the hypergeometric test using ShinyGO 0.77. Fold enrichment represents the percentage of genes in the analyzed list belonging to a pathway, divided by the corresponding percentage in the background (all) genes set. **(b)** Overlap between genes with increased expression in G0 cells in the absence of HIRA (*hip1*Δ) and the Clr4 histone H3 lysine 9 methyltransferase [11] (*p* value, hypergeometric test). **(c)** Venn diagrams summarizing numbers of differentially expressed genes in the *hip1*Δ mutant in G0 (−N 1 day) and during G0 exit (+N 90 mins). **(d)** a summary of the GO terms associated with two gene clusters, termed UP1 and UP2, which are robustly induced during exit from G0 [8]. **(e)** Box plots comparing the transcript abundance (log2 FPKM) of the indicated gene clusters in wild type and *hip1*Δ cells in G0 (−N 1 day) and during G0 exit (+N 90 mins) cells. **(f)** wild type and *hip1*Δ cells were grown to mid logarithmic phase in EMM and then suspended in EMM-N medium to induce quiescence. After 1 day in EMM-N medium a nitrogen source was resupplied by suspending cells in rich (YES) medium. At the indicated time points after nitrogen source restoration, RNA was prepared and *ura3*^*+*^ transcript (UP1 gene) levels were analyzed by RT-qPCR. Levels are scaled relative to the wild type time 0 sample and normalized using *sde2*^*+*^ transcript levels which remain constant in proliferating and nitrogen starved quiescent cells [7]. Data are the mean of three biological repeats. Error bars represent ±SEM. **(g)** RT-qPCR analysis of *mis3*^*+*^ (UP2 gene) transcript levels as described in (f).

As HIRA is required for quiescence, RNA-seq was used to compare the transcript profiles of wild type and *hip1*Δ G0 cells (nitrogen starved for 1 day). RNAs from a large number (*n* = 482) of genes were increased (>2-fold) in *hip1*Δ. GO term analysis of these genes (Figure 6a) revealed a significant enrichment of biological process terms associated with meiosis (e.g. meiotic drive, meiotic cell cycle processes and reproduction) which is consistent with a role for HIRA in suppressing meiosis-specific transcripts [39]. As is the case in proliferating cells [29], loss of HIRA function resulted in increased *Tf2* LTR retrotransposon RNA (GO term: DNA integration). Entry into G0 induces the *de novo* assembly of heterochromatin domains and disruption of heterochromatin results in differential gene expression and reduced survival in quiescence [11,12]. Genes that are de-repressed in G0 *hip1*Δ cells significantly overlapped with those that are upregulated in quiescent cells lacking the key heterochromatin histone H3 lysine 9 methyltransferase, Clr4 (Figure 6b) suggesting that HIRA contributes to transcriptional silencing in G0. We found that a smaller number (*n* = 250) of genes showed decreased expression (>2-fold) in G0 *hip1*Δ cells. GO terms analysis of these genes revealed terms related to the response to pheromone (*p* = 1.6 e-10) and the regulation of conjugation (*p* = 7.5 e-08). Overall, genes involved in sexual development are dysregulated in the absence of HIRA.

### HIRA is required for the induction of MBF-dependent genes during exit from G0

To understand why cells lacking HIRA do not efficiently exit quiescence and reenter the cell cycle, RNA-seq was also used to analyze the transcriptome of cells 90 min after the restoration of a nitrogen source. Analysis of the genes that are differentially expressed (>2-fold) in a *hip1*Δ mutant during exit from G0 is shown in Figure 6c and Table S2.

Previous studies have defined clusters of genes whose expression is induced when G0 cells transition back into the vegetative cell cycle [8]. In particular, the expression of genes in two clusters designated UP1 and UP2, is robustly and rapidly increased when a nitrogen source is re-supplied to G0 cells. Induction of these gene clusters is likely to be important for the increase in cell size (growth) that follows nitrogen restoration because these genes are associated with ribosome biogenesis, protein biosynthesis, translation factors, RNA polymerase I, RNA binding and RNA processing [8] (Figure 6d). As expected, the expression of the UP1 and UP2 gene clusters increased in wild type cells in response to nitrogen replenishment and importantly, a similar increase was also observed in *hip1*Δ cells (Figure 6e). This was confirmed by RT-qPCR analysis of *ura3*^+^ (UP1 gene) and *mis3*^+^ (UP2 gene) transcripts levels (Figure 6f,g).

These results are consistent with our findings that, after short-term G0 (1 day), most *hip1*Δ cells are capable of re-initiating growth (Figure 2c,d and Fig S2). Despite this, G0 *hip1*Δ cells are unable to efficiently reenter the vegetative cell cycle and so we hypothesized that HIRA may be required for the induction of genes required for the transition out of G0 and into S phase. Remodeling of the mitochondrial proteome and upregulation of genes involved in oxidative phosphorylation is one of the characteristics of T-cells undergoing exit from quiescence [40]. Notably, when we analyzed the 328 genes whose expression is significantly reduced in a *hip1*Δ mutant compared with wild-type cells exiting G0 (Figure 6c and Table S2) GO term analyses revealed enrichment for genes involved in “nucleoside monophosphate metabolic processes” (*p* = 6.09 e-04) (Table S3), many of which encode mitochondrial electron transport chain proteins involved in oxidative phosphorylation (Table S4).

**Figure 7.**
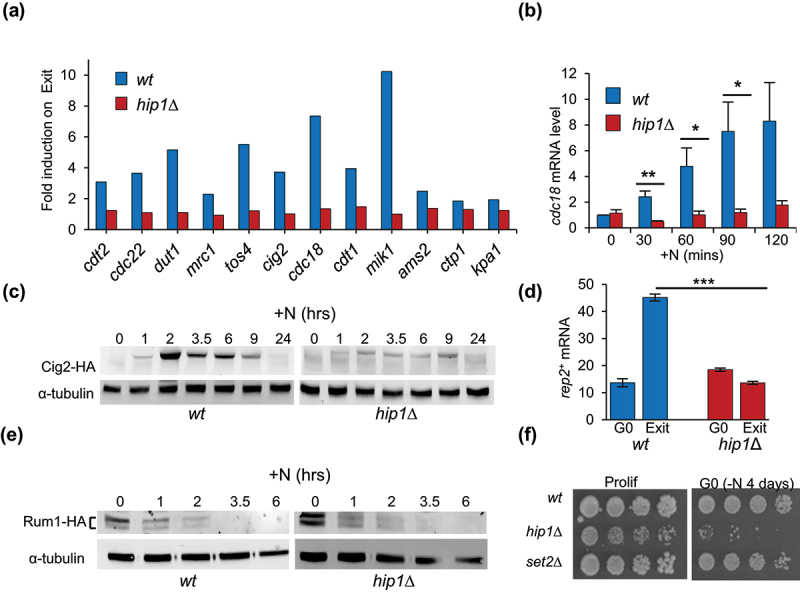
HIRA is required for the induction of MBF-dependent genes during exit from quiescence. **(a)** the fold increase in mRNA levels of MBF-dependent genes in wild type and *hip1*Δ cells during exit from G0 (90 minutes after the restoration of a nitrogen source) was determined by RNA-seq. Induction of all genes except *ctp1*^*+*^ was significantly reduced in *hip1*Δ (*p* < 0.01). **(b)** HIRA is required for the induction of *cdc18*^*+*^ during cell cycle reentry. *cdc18*^*+*^ mRNA levels following the replenishment of a nitrogen source to G0 cells were determined using RT-qPCR as described in fig 6F. Data are the mean of three biological repeats. Error bars represent ± SEM. (*p < 0.05, **p < 0.01; t-test). **(c)** HIRA is required for the accumulation of the Cig2 cyclin during cell cycle reentry. Wild type and *hip1*Δ cells expressing HA-tagged Cig2 (Cig2-HA) were grown to mid logarithmic phase in EMM and then resuspended in EMM-N medium to induce quiescence. After 1 day in EMM-N medium a nitrogen source was restored by suspending cells in rich (YES) medium. At the indicated time points after nitrogen source restoration, aliquots of the culture were taken and whole protein extracts were prepared. Protein extracts were analyzed by western blotting with anti-HA and anti-α-tubulin antibodies. α-tubulin serves as a loading control. Data are representative of three biological repeats. **(d)** HIRA is required for the induction of *rep2*^*+*^ during reentry into the cell cycle. The abundance of *rep2*^*+*^ transcripts (FPKM) in wild type and *hip1*Δ G0 cells (−N for 1 day) and in cells undergoing exit from G0 (90 min after the restoration of a nitrogen source) was determined by RNA-seq. Error bars represent ± SEM. (***p < 0.001; t-test). **(e)** HIRA is not required for the removal of Rum1 during cell cycle reentry. Rum1-HA levels in wild type and *hip1*Δ following the restoration of a nitrogen source to G0 cells were determined by western blotting as described in (c). Data are representative of four biological repeats. **(f)** loss of Set2 does not result in a rapid loss in proliferative capacity in G0. The indicated strains (wild type, *hip1*Δ and *set2*Δ), were grown to mid logarithmic phase in EMM medium (Prolif) and then suspended in EMM-N medium for 4 days (−N 4 days). Cultures were subjected to five-fold serial dilution, printed onto YES agar and incubated at 30°C for 3–4 days to allow colonies to form.

We also found that targets of Cdc10 were significantly overrepresented (*p* = 7.7 e-06) in the 328 genes whose expression is reduced in *hip1*Δ during G0 exit. Cdc10 is a core subunit of the MBF transcription factor complex, the functional equivalent of mammalian E2F, which activates gene expression that is critical for S phase [41]. Analysis of the RNA-seq data revealed that the expression of all MBF-target genes except *ctp1*^*+*^ was significantly impaired (*p* < 0.01) in the *hip1Δ* mutant (Figure 7a). This finding was confirmed using RT-qPCR to monitor the expression of the key MBF-target *cdc18*^*+*^ (a homolog of human *CDC6*) following nitrogen source replenishment (Figure 7b). Furthermore, accumulation of the major G1-S cyclin, Cig2 was also severely reduced in *hip1*Δ cells (Figure 7c). Therefore, HIRA is required for the induction of MBF-dependent genes during reentry into the cell cycle from G0.

The finding that HIRA is necessary for the induction of the MBF regulon during exit from quiescence, prompted us to examine whether it affected the expression of genes encoding the components of MBF. Analysis of the RNA-seq data revealed that HIRA had only a minor impact on the expression of MBF core subunit (*res1*^*+*^, *res2*^*+*^, and *cdc10*^+^) and co-repressor (*nrm1*^+^ and *yox1*^+^) genes (Table S5). In contrast, the expression of the co-activator *rep2*^+^ was significantly affected in *hip1*Δ. As previously observed [42], the expression of *rep2*^*+*^ was robustly induced as wild type cells exit G0 but we found that this was abolished in the *hip1*Δ mutant (Figure 7d). This is important as the accumulation of Rep2 is believed to underpin the potential of MBF to activate transcription [43].

Next, we investigated the levels of the cyclin-dependent kinase inhibitor (CKI) Rum1 which is present at high levels in G0 cells and blocks cell cycle entry. Upon restoration of a nitrogen source, Rum1 is degraded which leads to progression into S phase [8]. Western blotting revealed that Rum1 was present at high levels in wild type and *hip1*Δ G0 cells and decreased drastically in both strains within hours of nitrogen restoration (Figure 7e) indicating HIRA acts downstream of Rum1 removal.

**Figure 8.**
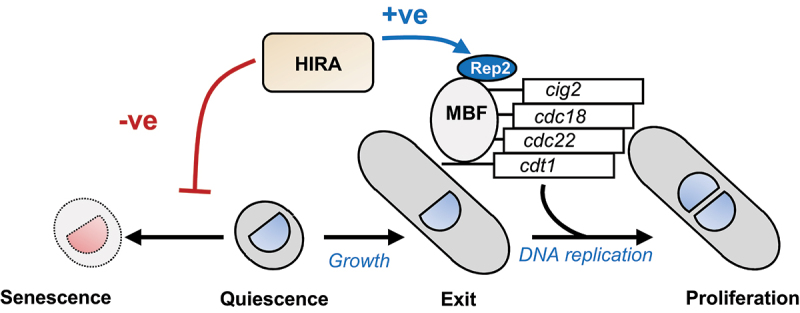
Model summarizing the roles of HIRA in (G0) quiescence. HIRA mediates efficient exit from quiescence. HIRA is required for the induction of the MBF transcription factor coactivator subunit Rep2 during exit from G0. As a result, induction of MBF-target genes such as *cdc18*^*+*^, *cdt1*^*+*^ and *cdc22*^*+*^, which are required for entry into S phase are dependent upon HIRA. In addition, HIRA prevents the premature onset of senescence. G0 cells lacking HIRA rapidly progress to a permanent cell cycle arrest in which they retain metabolic activity but can no longer re-initiate growth (elongate) and resume proliferation in response to restoration of a nitrogen source. Overall, the longevity and reversibility of quiescence are dependent upon HIRA.

G0 cells lacking HIRA function rapidly progress to a permanently arrested “senescent” state. To determine whether an inability to induce MBF-dependent gene expression is sufficient to cause this phenotype, we analyzed a *set2*Δ mutant. Set2 is a histone H3K36 methyl transferase that is also required for the induction of MBF genes during G0 exit and accordingly, *set2*Δ mutants also have delayed entry into S phase [44]. However, nitrogen-starved *set2*Δ cells did not exhibit a pronounced loss of proliferative capacity (Figure 7f) indicating that defects in the induction of MBF genes do not necessarily result in an irreversible cell cycle arrest. Taken together, our data suggest that the HIRA complex has multiple roles in quiescence. Firstly, HIRA is required for the efficient induction of MBF genes and therefore exit from G0 and secondly, it prevents the premature onset of senescence (Figure 8).

## Discussion

Here we demonstrate that the HIRA complex is required for the longevity and reversibility of quiescence in *S. pombe*. It is noteworthy that a previous high-throughput genetic analysis of chromatin regulators did not identify a major role for HIRA in quiescence [10]. Indeed, HIRA mutant strains were found to have only a modest reduction in survival in G0 relative to wild type. However, this high-throughput screen used propidium iodide staining and flow cytometry to distinguish between “live” and “dead” cells and thus measure survival in quiescence. Our findings also indicate that *hip1*Δ mutants retain metabolic activity for a prolonged period in G0 (Figure 2f,g) and in this respect are consistent with the high-throughput screen. However, we find that despite retaining metabolic activity, HIRA-deficient G0 cells rapidly lose the ability to resume proliferation and so lack a key hallmark of quiescence. Our studies highlight the utility of measuring proliferative capacity when assessing quiescence.

After a short G0, cells lacking HIRA can resume growth but are defective in their ability to reenter the vegetative cell cycle which correlates with a failure to induce the expression of *rep2*^*+*^ and as a result, MBF target genes. This finding was unexpected because HIRA is not necessary for the proper expression of MBF target genes at G1/S in cycling cells [15]. Rep2 is an unstable protein whose levels oscillate during the cell cycle with a peak that is co-incident with MBF gene expression and Rep2 accumulation is believed to be critical for MBF to activate transcription from target promoters [43]. Rep2 levels also control entry in G0 as overexpression results in an aberrant G2/M arrest in response to nitrogen starvation whereas *rep2*^*+*^ deletion results in premature entry into G0 [42]. A role for HIRA in the induction of gene expression was also unexpected as transcriptomic analysis suggested that it predominantly functions as a transcriptional repressor in cycling fission yeast cells [29]. Indeed, impairing HIRA function results in the widespread upregulation of repressed genes and, consistent with a role as a nucleosome assembly factor, a global reduction in nucleosome levels [29,35]. However, histone chaperones function in a context-dependent manner and are also capable of facilitating nucleosome removal. For example, nucleosome eviction from specific *S. pombe* stress-responsive genes is impaired in the absence of HIRA and the induction of these genes is HIRA-dependent [45]. It is possible that HIRA may facilitate the induction of *rep2*^*+*^ and, as a result, MBF-regulated genes by a similar mechanism. Future work to characterize the changes in nucleosomal occupancy and positioning that occur in G0 cells lacking HIRA may help address this question.

That HIRA function impacts upon MBF-dependent gene expression is intriguing given that G1/S transcription factors play key roles in quiescence regulation. In *S. cerevisiae* the G1/S transcriptional program is controlled by MBF (composed of homologs of *S. pombe* MBF subunits Cdc10 and Res1/2) and a closely related factor called SBF. The entry of *S. cerevisiae* cells into a quiescent (Q cell) state requires the stable but reversible repression of G1/S genes. This is mediated by Msa1 and Msa2 which recruit an HDAC complex (Rpd3/Sin3/Sds3) to MBF and SBF [46]. Msa1 and Msa2 are therefore functionally equivalent to the DREAM (E2F4/DP/RBL/MuvB) complex of metazoans that promotes quiescence by recruiting HDAC1 to E2F-activated genes [47]. Whether the HIR complex is required for the reversal of Msa1/2-mediated repression during quiescence exit in *S. cerevisiae* remains to be determined. *S. pombe* does not have homologs of Msa1/2 and MBF co-repressors Nrm1 and Yox1 are not required for entry into quiescence [10]. However, loss of Nrm1 and Yox1 leads to reduced survival in G0 suggesting that the maintenance of quiescence requires the suppression of MBF activity [10].

In addition to being necessary for efficient exit from G0, HIRA also prevents the premature onset of senescence. G0 cells lacking HIRA function rapidly progress to a state in which they can no longer resume growth in response to nitrogen source restoration (Figure 2c). Importantly, this is not observed in a *set2Δ* mutant (Figure 7f), suggesting that defects in MBF-dependent gene expression alone do not account for this phenotype. The reason why impaired HIRA function results in a rapid progression to an irreversible cell cycle arrest remains to be determined although it is possible that this reflects a role in the regulation of transcription. The expression of genes related to sexual development (e.g. conjugation and meiosis) are dysregulated in cells lacking HIRA and this inappropriate expression may adversely affect the maintenance of a quiescent state. In *S. cerevisiae* entry into quiescence is accompanied by a global transcriptional shut-off, which is dependent upon the targeting of the Rpd3 HDAC to at least half of all gene promoters [6]. A drastic shrinkage in the transcriptome is also observed in quiescent *S. pombe* cells [7]. This transcriptional reprogramming is at least partly dependent upon the *de novo* formation of RNAi-dependent heterochromatin and accordingly mutations in key heterochromatin regulators such as the H3K9 methyltransferase Clr4, result in altered expression of a large set of genes and reduced survival in quiescence [11,12]. The integrity of pericentric and *mat* locus heterochromatin is HIRA-dependent in proliferating cells [15] and we find a significant overlap between genes that are differentially expressed during G0 in *hip1*Δ and *clr4*Δ backgrounds (Figure 6b). Therefore, the reduced longevity of quiescence in HIRA mutants may be partially attributable to heterochromatin dysregulation. Although we note that, when compared to the loss of heterochromatin regulators, the inactivation of HIRA results in a much more rapid loss of proliferative potential. In the case of the RNAi mutant, *dcr1*Δ the identification of suppressor mutations provided important insights into the basis of the G0 defect [12]. A similar approach may prove useful for further defining the roles of HIRA in quiescence.

## Supplementary Material

Supplemental Material

## Data Availability

Authors agree to make data and materials supporting the results or analyses presented in their paper available upon reasonable request. RNA-seq data have been submitted to GEO (accession number: GSE129599).
