# Supplementary material for: The longevity and reversibility of quiescence in Schizosaccharomyces pombe are dependent upon the HIRA histone chaperone
Source: Cell Cycle. 2023 Aug 27;22(17):1921–36. doi: 10.1080/15384101.2023.2249705 (PMC10599175; doi:10.1080/15384101.2023.2249705)
Supplement: Supplemental Material [file KCCY_A_2249705_SM9609.zip › Fig S1.pptx]

## Slide 1
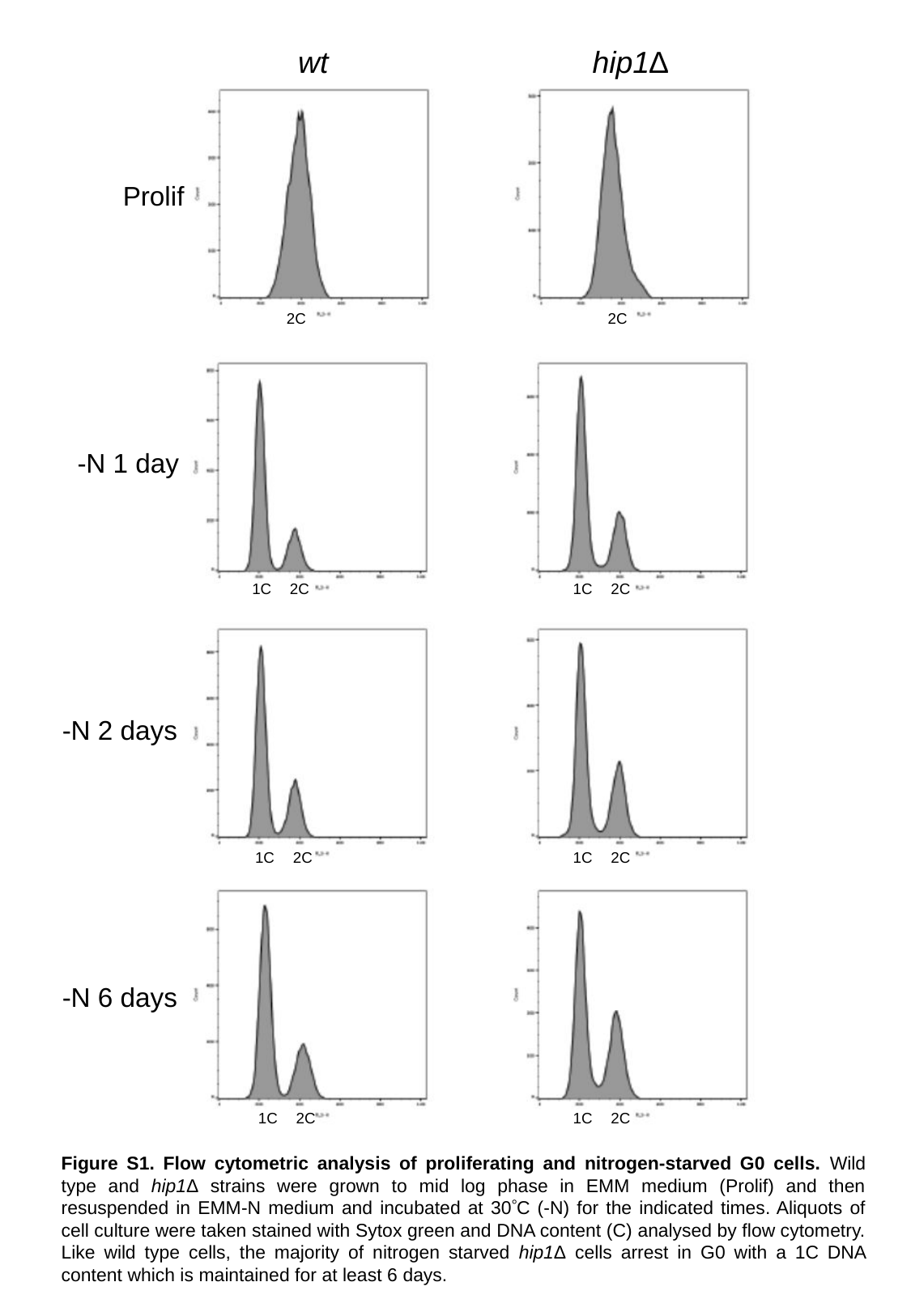

wt
hip1∆
Prolif
2C
2C
-N 1 day
1C
2C
1C
2C
-N 2 days
1C
2C
1C
2C
-N 6 days
1C
2C
1C
2C
Figure S1. Flow cytometric analysis of proliferating and nitrogen-starved G0 cells. Wild type and hip1Δ strains were grown to mid log phase in EMM medium (Prolif) and then resuspended in EMM-N medium and incubated at 30°C (-N) for the indicated times. Aliquots of cell culture were taken stained with Sytox green and DNA content (C) analysed by flow cytometry. Like wild type cells, the majority of nitrogen starved hip1Δ cells arrest in G0 with a 1C DNA content which is maintained for at least 6 days.
