# Supplementary material for: The longevity and reversibility of quiescence in Schizosaccharomyces pombe are dependent upon the HIRA histone chaperone
Source: Cell Cycle. 2023 Aug 27;22(17):1921–36. doi: 10.1080/15384101.2023.2249705 (PMC10599175; doi:10.1080/15384101.2023.2249705)
Supplement: Supplemental Material [file KCCY_A_2249705_SM9609.zip › Fig S2.pptx]

## Slide 1
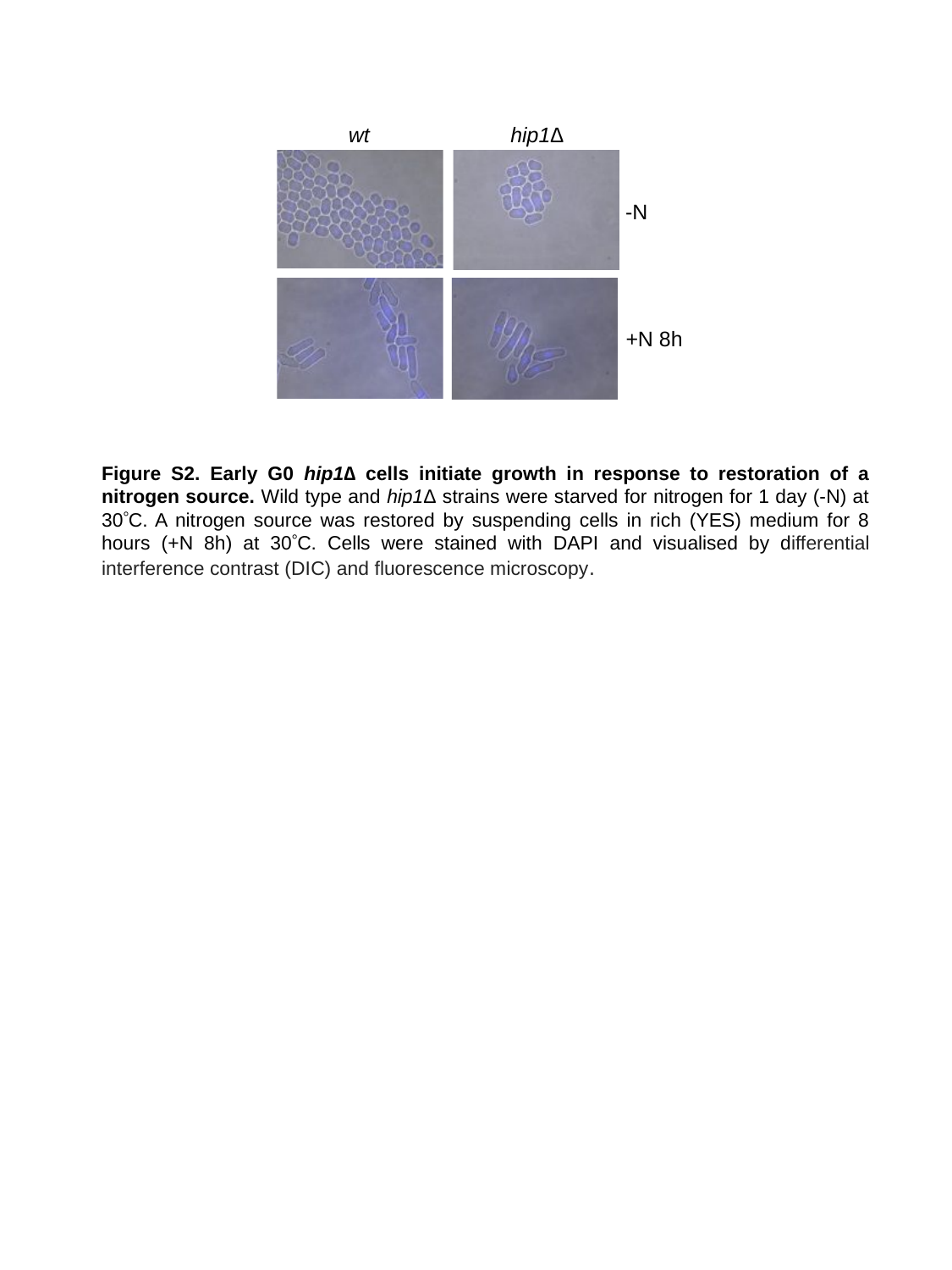

wt
hip1∆
-N
+N 8h
Figure S2. Early G0 hip1∆ cells initiate growth in response to restoration of a nitrogen source. Wild type and hip1Δ strains were starved for nitrogen for 1 day (-N) at 30°C. A nitrogen source was restored by suspending cells in rich (YES) medium for 8 hours (+N 8h) at 30°C. Cells were stained with DAPI and visualised by differential interference contrast (DIC) and fluorescence microscopy.
