# Supplementary material for: The longevity and reversibility of quiescence in Schizosaccharomyces pombe are dependent upon the HIRA histone chaperone
Source: Cell Cycle. 2023 Aug 27;22(17):1921–36. doi: 10.1080/15384101.2023.2249705 (PMC10599175; doi:10.1080/15384101.2023.2249705)
Supplement: Supplemental Material [file KCCY_A_2249705_SM9609.zip › Fig S3.pptx]

## Slide 1
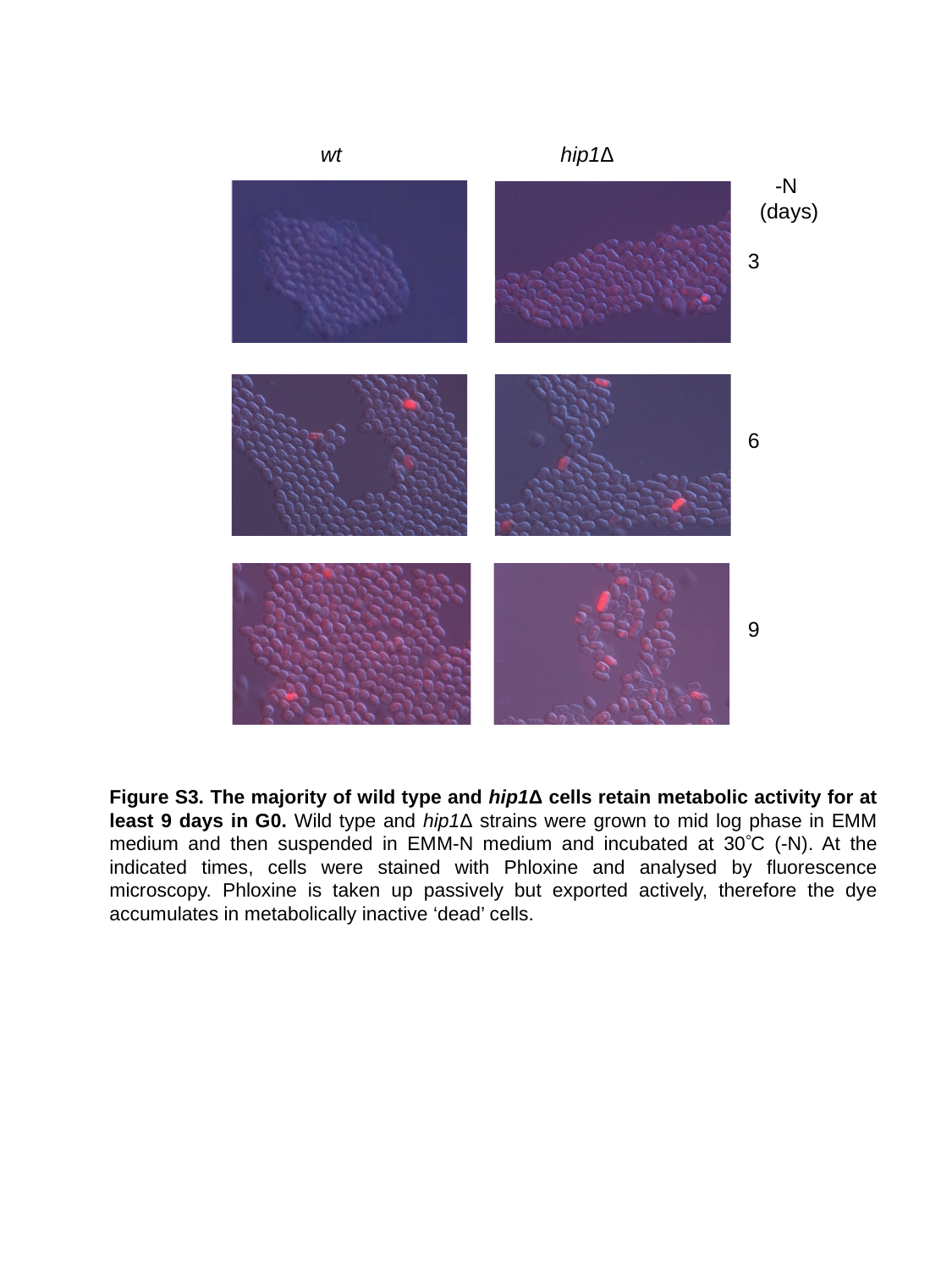

wt
hip1∆
-N
(days)
3
6
9
Figure S3. The majority of wild type and hip1Δ cells retain metabolic activity for at least 9 days in G0. Wild type and hip1Δ strains were grown to mid log phase in EMM medium and then suspended in EMM-N medium and incubated at 30°C (-N). At the indicated times, cells were stained with Phloxine and analysed by fluorescence microscopy. Phloxine is taken up passively but exported actively, therefore the dye accumulates in metabolically inactive ‘dead’ cells.
