# Supplementary material for: The longevity and reversibility of quiescence in Schizosaccharomyces pombe are dependent upon the HIRA histone chaperone
Source: Cell Cycle. 2023 Aug 27;22(17):1921–36. doi: 10.1080/15384101.2023.2249705 (PMC10599175; doi:10.1080/15384101.2023.2249705)
Supplement: Supplemental Material [file KCCY_A_2249705_SM9609.zip › Fig S4.pptx]

## Slide 1
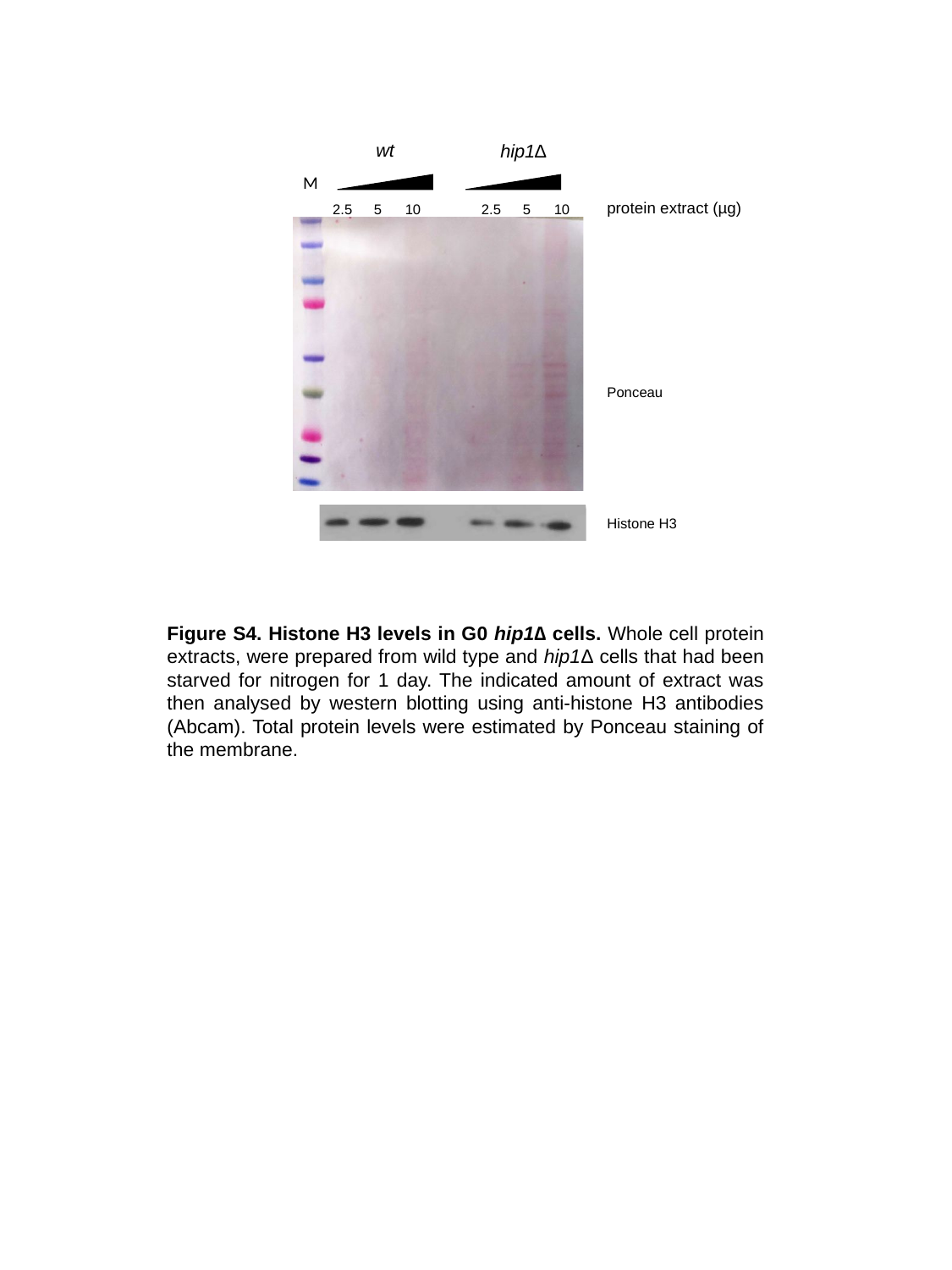

wt
hip1∆
M
protein extract (µg)
2.5
5
10
2.5
5
10
Ponceau
Histone H3
Figure S4. Histone H3 levels in G0 hip1∆ cells. Whole cell protein extracts, were prepared from wild type and hip1Δ cells that had been starved for nitrogen for 1 day. The indicated amount of extract was then analysed by western blotting using anti-histone H3 antibodies (Abcam). Total protein levels were estimated by Ponceau staining of the membrane.
