# Supplementary material for: The longevity and reversibility of quiescence in Schizosaccharomyces pombe are dependent upon the HIRA histone chaperone
Source: Cell Cycle. 2023 Aug 27;22(17):1921–36. doi: 10.1080/15384101.2023.2249705 (PMC10599175; doi:10.1080/15384101.2023.2249705)
Supplement: Supplemental Material [file KCCY_A_2249705_SM9609.zip › Fig S5.pptx]

## Slide 1
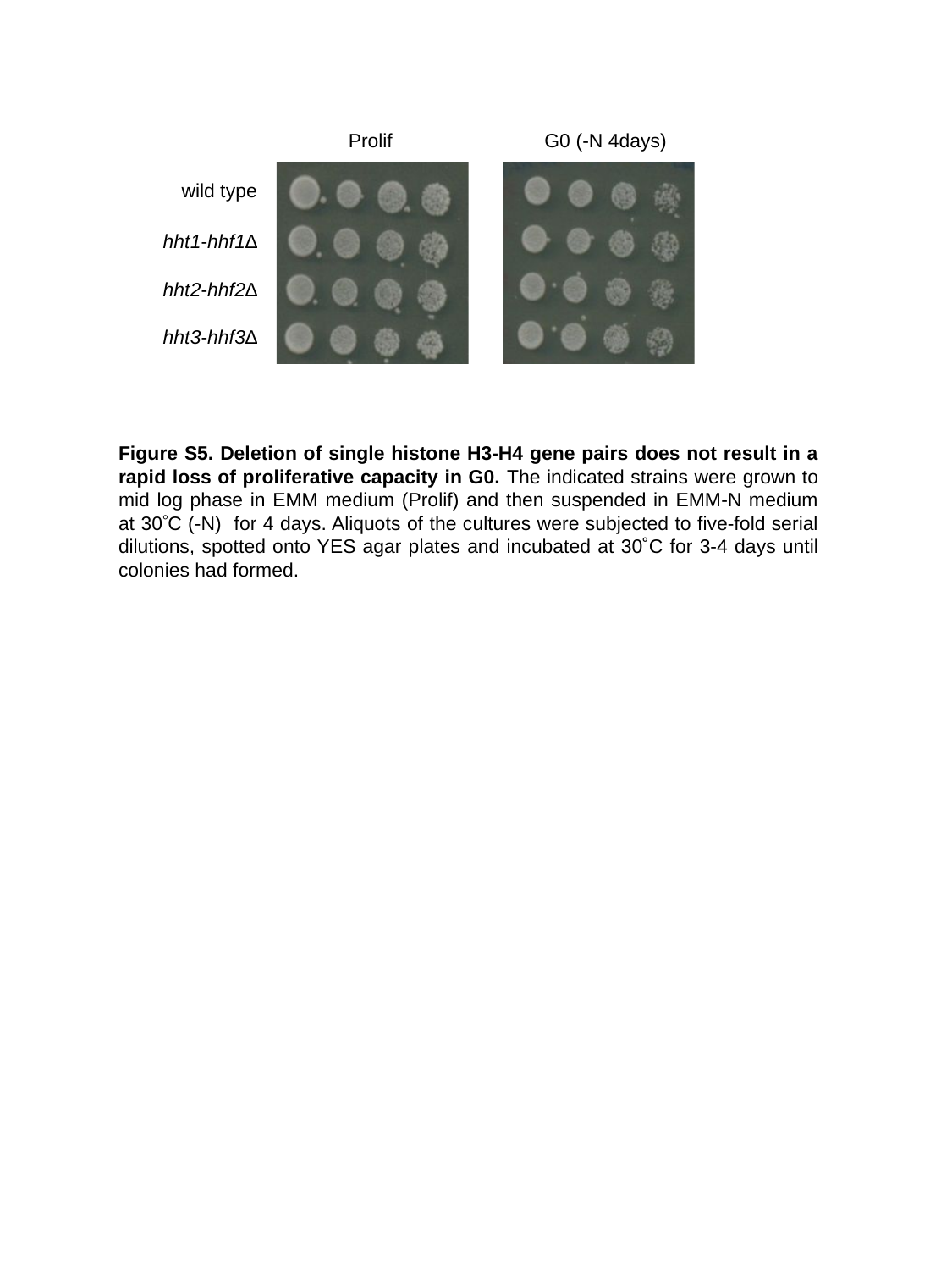

Prolif
G0 (-N 4days)
wild type
hht1-hhf1∆
hht2-hhf2∆
hht3-hhf3∆
Figure S5. Deletion of single histone H3-H4 gene pairs does not result in a rapid loss of proliferative capacity in G0. The indicated strains were grown to mid log phase in EMM medium (Prolif) and then suspended in EMM-N medium at 30°C (-N) for 4 days. Aliquots of the cultures were subjected to five-fold serial dilutions, spotted onto YES agar plates and incubated at 30˚C for 3-4 days until colonies had formed.
