# Supplementary material for: The longevity and reversibility of quiescence in Schizosaccharomyces pombe are dependent upon the HIRA histone chaperone
Source: Cell Cycle. 2023 Aug 27;22(17):1921–36. doi: 10.1080/15384101.2023.2249705 (PMC10599175; doi:10.1080/15384101.2023.2249705)
Supplement: Supplemental Material [file KCCY_A_2249705_SM9609.zip › Fig S6.pptx]

## Slide 1
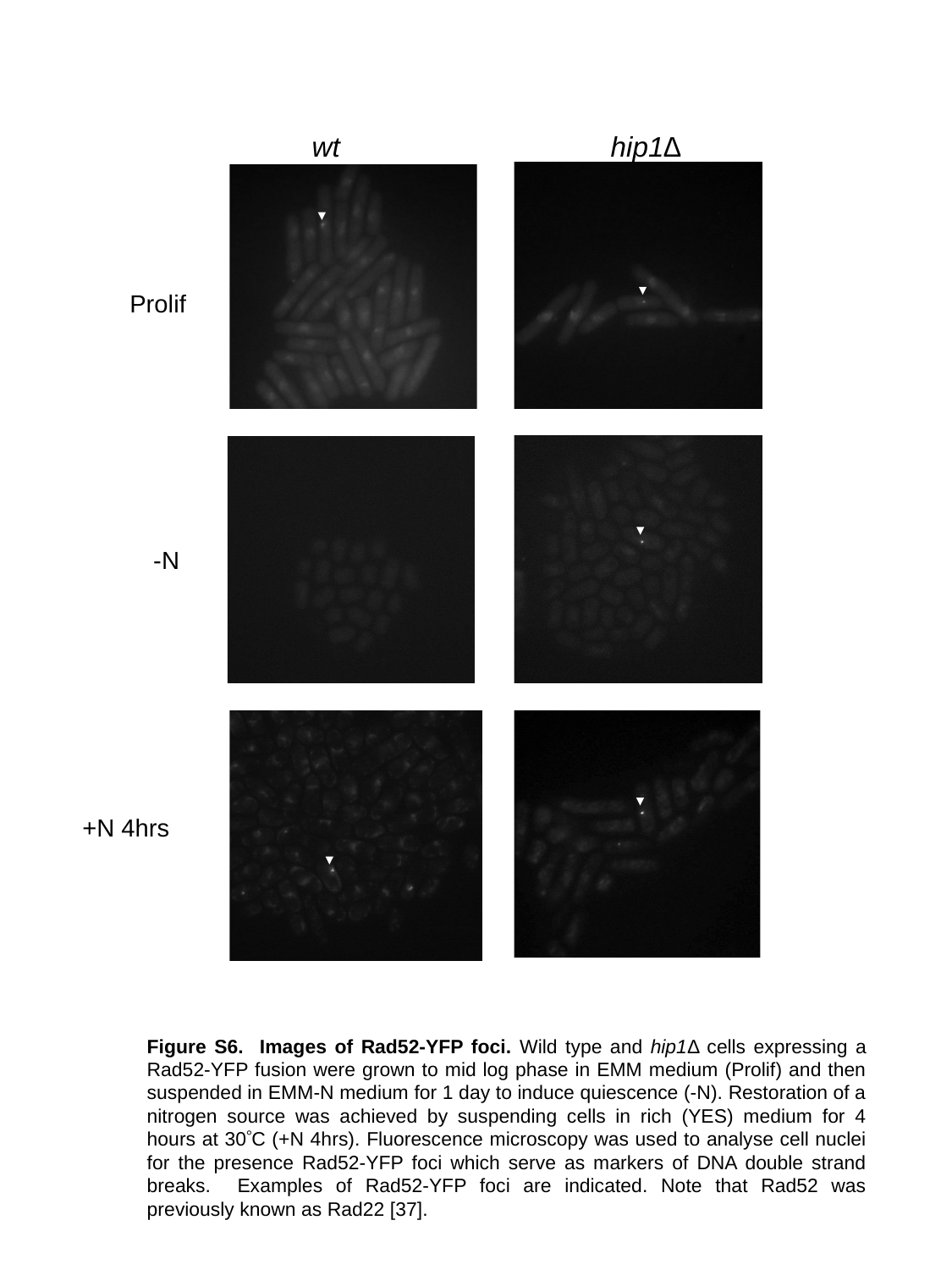

hip1∆
wt
Prolif
-N
+N 4hrs
Figure S6. Images of Rad52-YFP foci. Wild type and hip1Δ cells expressing a Rad52-YFP fusion were grown to mid log phase in EMM medium (Prolif) and then suspended in EMM-N medium for 1 day to induce quiescence (-N). Restoration of a nitrogen source was achieved by suspending cells in rich (YES) medium for 4 hours at 30°C (+N 4hrs). Fluorescence microscopy was used to analyse cell nuclei for the presence Rad52-YFP foci which serve as markers of DNA double strand breaks. Examples of Rad52-YFP foci are indicated. Note that Rad52 was previously known as Rad22 [37].
