# Supplementary material for: The longevity and reversibility of quiescence in Schizosaccharomyces pombe are dependent upon the HIRA histone chaperone
Source: Cell Cycle. 2023 Aug 27;22(17):1921–36. doi: 10.1080/15384101.2023.2249705 (PMC10599175; doi:10.1080/15384101.2023.2249705)
Supplement: Supplemental Material [file KCCY_A_2249705_SM9609.zip › Table S1.docx]

**Table S1. Strains used in this study.**

| **Strain** | **Genotype** | **Source/Ref** |
| --- | --- | --- |
| 972 | *h^-^* | Lab stock |
| CsG20 | *h^-^ hip1::ura4^+^ ura4-D18* | This study |
| CsG21 | *h^-^ slm9::ura4^+^ ura4-D18* | This study |
| CsG207 | *h^-^ hip3::ura4^+^ ura4-D18* | This study |
| CsG213 | *h^+^ hip4::ura4^+^ ura4-D18* | This study |
| JT268 | *h^-^ atg8-NGFP* | [[1](#_ENREF_1)] |
| CsG124 | *h^-^ atg8-NGFP hip1::kanMX* | This study |
| CsG22 | *h^-^ hht1/hhf1::ura4^+^ ura4-D18* | This study |
| CsG23 | *h^-^ hht2/hhf2::ura4^+^ ura4-D18* | This study |
| CsG24 | *h^-^ hht3/hhf3::ura4^+^ ura4-D18* | This study |
| CsG14 | *h^-^ hht1/hhf1::ura4^+^ hht2/hhf2::ura4^+^ otr1R (Sph1):ade6^+^* | This study |
| CsG15 | *h^+^ hht2/hhf2::ura4^+^ hht3.3/hhf3::ura4^+^ otr1R (Sph1):ade6^+^* | This study |
| CsG243 | *h^-^ hht2/hhf12::ura4^+^ hip1::kanMX* | This study |
| CsG25 | *h^+^ hip1-HBD(ura4^+^)* | This study |
| CsG1 | *h^-^ rad52-YFP(kanMX)*^*^ | This study |
| CsG2 | *h^-^ rad52-YFP(kanMX) hip1::ura4^+^* | This study |
| CsG328 | *h^-^ cig2-HA hip1::kanMX* | This study |
| PN1399 | *h^-^ cig2-HA* | [[2](#_ENREF_2)] |
| CsG300 | *h^-^ rum1-HA* | S. Moreno |
| CsG301 | *h^-^ rum1-HA hip1::kanMX* | This study |
| CsG69 | *h^-^  nap1::kanMX* | This study |
| CsG66 | *h^-^  nap2::kanMX* | This study |
| CsG67 | *h^-^  pcf2::kanMX* | This study |
| CsG101 | *h^-^  rtt106::kanMX* | This study |
| CsG39 | *h^-^ asf1-33-13myc(kanMX)* | This study |
| SW1022 | *h^+^ pob3::natMX* | This study |
| CsG141 | *h^-^  set2::kanMX* | This study |

* Also known as *rad22-YFP* [[3](#_ENREF_3)]

**References**

1. Mukaiyama, H., et al., *Autophagy-deficient Schizosaccharomyces pombe mutants undergo partial sporulation during nitrogen starvation.* Microbiology, 2009. **155**(Pt 12): p. 3816-26.

2. Martin-Castellanos, C., K. Labib, and S. Moreno, *B-type cyclins regulate G1 progression in fission yeast in opposition to the p25rum1 cdk inhibitor.* EMBO J, 1996. **15**(4): p. 839-49.

3. Noguchi, E., et al., *Swi1 prevents replication fork collapse and controls checkpoint kinase Cds1.* Mol Cell Biol, 2003. **23**(21): p. 7861-74.
