# Supplementary material for: The longevity and reversibility of quiescence in Schizosaccharomyces pombe are dependent upon the HIRA histone chaperone
Source: Cell Cycle. 2023 Aug 27;22(17):1921–36. doi: 10.1080/15384101.2023.2249705 (PMC10599175; doi:10.1080/15384101.2023.2249705)
Supplement: Supplemental Material [file KCCY_A_2249705_SM9609.zip › Table S2.docx]

**Table S2. Genes differentially expressed (> 2 fold) in *hip1*Δ during quiescence (G0) and exit from quiescence**

| Decreased in G0 | Decreased in Exit | Decreased in G0 & Exit | Increased in G0 | Increased in Exit | Increased in G0 & Exit |
| --- | --- | --- | --- | --- | --- |
| SPAC5H10.03 | SPCC330.06c | SPBC4C3.09 | SPBC460.03 | SPCC320.05 | SPCC1884.02 |
| SPAC24B11.06c | SPAC806.03c | SPAC977.11 | SPBC460.05 | SPBC609.01 | SPCC757.02c |
| SPAC24B11.07c | SPCC16C4.22 | SPMTR.04 | SPAC31A2.05c | SPAC977.17 | SPAC212.02 |
| SPAC806.11 | SPAC630.12 | SPCC1020.08 | SPAC31A2.06 | SPAC11D3.08c | SPAC977.02 |
| SPBC1105.02c | SPBC16D10.11c | SPBC887.07 | SPBC16D10.04c | SPAC5H10.07 | SPAC977.08 |
| SPCC24B10.10c | SPCC645.12c | SPCPB16A4.03c | SPBP8B7.05c | SPCC594.06c | SPBC4C3.08 |
| SPCC24B10.12 | SPAC1D4.10 | SPCC550.11 | SPCC1322.07c | SPAC24B11.05 | SPAC977.15 |
| SPCC4B3.02c | SPAC1296.02 | SPAC3H8.07c | SPAC22G7.11c | SPBC460.01c | SPAC1F8.01 |
| SPCC1322.10 | SPBC1604.07 | SPAC1D4.08 | SPCC663.14c | SPCC1672.04c | SPAC1F8.02c |
| SPAC22F3.12c | SPBC1677.03c | SPAC1D4.13 | SPBCPT2R1.02 | SPCC1183.12 | SPCC1235.17 |
| SPAC1687.01 | SPBC16A3.12c | SPBC21C3.17c | SPAC23C4.18c | SPBPB7E8.01 | SPAC11D3.10 |
| SPAC4C5.03 | SPAC821.10c | SPBC23E6.10c | SPCC576.19c | SPAC1751.01c | SPBC460.04c |
| SPBC1685.01 | SPBC1683.11c | SPBC32C12.02 | SPBPB10D8.07c | SPBC1105.10 | SPCC364.05 |
| SPBC354.08c | SPAC22A12.07c | SPBC3B9.19 | SPAC30D11.02c | SPAC13C5.04 | SPCC970.11c |
| SPAPB1A10.08 | SPBC428.11 | SPBC16A3.15c | SPCC126.13c | SPAC13C5.06c | SPMIT.11 |
| SPCC16C4.20c | SPAC23H4.09 | SPAC56F8.07 | SPAC22A12.09c | SPBC1105.14 | SPCC1020.05 |
| SPCC16C4.17 | SPBC649.02 | SPAC56F8.09 | SPCC794.06 | SPCC24B10.19c | SPCC1393.07c |
| SPCC2H8.02 | SPAP14E8.02 | SPAC22A12.16 | SPBC1685.04 | SPCC24B10.20 | SPBC1105.13c |
| SPCC24B10.03 | SPCC16A11.14 | SPCC330.03c | SPAP27G11.15 | SPCPB16A4.07 | SPAC2F7.10 |
| SPAC4G9.20c | SPBC4.03c | SPAC23H4.04 | SPAC664.12c | SPCPB16A4.06c | SPCC645.05c |
| SPBC3D6.02 | SPBC725.15 | SPBC146.08c | SPBC530.02 | SPCC4E9.01c | SPBC21C3.01c |
| SPAC32A11.01 | SPAC18G6.04c | SPBC409.14c | SPBC36.01c | SPCC645.02 | SPCC1322.06 |
| SPCC417.02 | SPCC1322.09 | SPAC32A11.02c | SPAC17H9.02 | SPAC22F3.12c | SPCC1281.04 |
| SPBC19C2.07 | SPAC6C3.09 | SPBC19C2.03 | SPBC337.11 | SPBC23E6.03c | SPAC22F3.02 |
| SPCC297.04c | SPAC17G8.03c | SPCC576.13 | SPBC1709.01 | SPBC1604.17c | SPCC622.13c |
| SPCC576.01c | SPCC584.13 | SPAC3G9.10c | SPCC338.17c | SPCC622.01c | SPCC13B11.03c |
| SPAC31G5.10 | SPBC83.04 | SPAC3G9.06 | SPBC27B12.01c | SPAC22G7.04 | SPCC417.12 |
| SPCC1827.06c | SPBC83.05 | SPAC3G9.02 | SPCC11E10.08 | SPCC622.12c | SPCC1450.08c |
| SPBC29A3.08 | SPBC27.01c | SPBC17G9.06c | SPCC777.11 | SPAC4G8.03c | SPCC1223.12c |
| SPCC794.04c | SPBC19C2.15c | SPCC18B5.04 | SPCC663.11 | SPBC215.03c | SPBP4G3.03 |
| SPCC794.12c | SPBC2F12.11c | SPAC19G12.05 | SPBC21B10.06c | SPCC584.16c | SPBPB2B2.08 |
| SPAC17A2.08c | SPCC290.04 | SPBC1778.01c | SPCC285.16c | SPCC1753.03c | SPBC1348.01 |
| SPBC25B2.02c | SPBC16E9.05 | SPBC25H2.10c | SPCC737.08 | SPCC162.03 | SPBC1348.12 |
| SPAC15E1.10 | SPAC3G9.04 | SPAC30C2.02 | SPBC28E12.05 | SPAC22H12.01c | SPBPB21E7.01c |
| SPBC25B2.08 | SPBC1711.18 | SPBC1105.18c | SPBC18E5.03c | SPBC16G5.02c | SPCC1739.15 |
| SPAC1071.10c | SPAPB1E7.09 | SPAC19B12.02c | SPAC3G9.01 | SPAC222.19 | SPBPB21E7.10 |
| SPBC32F12.15 | SPAC16E8.04c | SPAPB8E5.05 | SPCC330.07c | SPBC543.05c | SPCC576.02 |
| SPAC19G12.10c | SPCC1672.05c | SPAPB8E5.09 | SPAC6G9.10c | SPAC222.08c | SPBPB10D8.01 |
| SPCC162.10 | SPAC15A10.10 | SPCC548.06c | SPBC19G7.07c | SPBC1289.10c | SPCC576.17c |
| SPAC11H11.04 | SPAC15E1.05c | SPAC4D7.14 | SPAC2E1P5.02c | SPBC1289.15 | SPBC359.04c |
| SPBP8B7.02 | SPAC27E2.03c | SPBC543.02c | SPAC328.01c | SPCC1223.13 | SPCC1620.13 |
| SPBC13G1.15c | SPAC19G12.15c | SPBPB8B6.06c | SPCC1682.03c | SPBC8E4.03 | SPAC56F8.12 |
| SPAPB8E5.03 | SPCC16A11.01 | SPCC1223.11 | SPCC1393.10 | SPBC8E4.12c | SPCC1919.11 |
| SPCC70.03c | SPCC16A11.17 | SPCC1906.01 | SPBC2A9.03 | SPBC8E4.02c | SPCC965.12 |
| SPCC736.15 | SPBC1703.13c | SPBPJ4664.03 | SPAC25B8.06c | SPCC737.09c | SPCP1E11.03 |
| SPCC594.02c | SPCC24B10.13 | SPBC409.15 | SPCC11E10.07c | SPBPB21E7.11 | SPBC800.07c |
| SPBC1683.01 | SPBC2D10.11c | SPAC1705.03c | SPCC188.13c | SPBC1683.02 | SPCP20C8.02c |
| SPBC1683.06c | SPCC4B3.20 | SPBC21B10.05c | SPCC417.04 | SPAC22A12.02c | SPCC320.07c |
| SPAC18B11.04 | SPCC645.11c | SPBC16E9.01c | SPAC1093.06c | SPBC1198.14c | SPAC57A10.06 |
| SPCC1442.01 | SPBC776.04 | SPBC1711.02 | SPCC1450.07c | SPCC1919.12c | SPCC1235.12c |
| SPCPB1C11.03 | SPCC162.01c | SPBC15C4.03 | SPBC16D10.06 | SPBC660.09 | SPCC794.02 |
| SPAC4G8.13c | SPCC417.04 | SPBC24C6.06 | SPCC1919.06c | SPCC965.09 | SPBC106.09 |
| SPAC1565.01 | SPAC12B10.04 | SPAC22H10.12c | SPAPB8E5.10 | SPCC965.11c | SPBC582.10c |
| SPAC19E9.03 | SPAC458.07 | SPBC4F6.14 | SPCC1494.10 | SPCC1494.08c | SPAC167.08 |
| SPAC20G8.04c | SPBC887.14c | SPAC513.03 | SPAC1952.10c | SPCC70.12c | SPCC5E4.04 |
| SPBC83.15 | SPBC31F10.07 | SPBP19A11.01 | SPCC1529.01 | SPCC70.09c | SPBC713.14c |
| SPAC3C7.11c | SPAC4F10.18 | SPAC688.12c | SPCC895.05 | SPAC10F6.05c | SPAC9G1.14 |
| SPBC15C4.06c | SPCC126.12 | SPAC3G9.09c | SPAC29B12.07 | SPCC330.01c | SPCC1020.01c |
| SPAC4G9.22 | SPBC211.04c | SPBC25H2.04c | SPCC4B3.18 | SPBC1773.09c | SPCC1259.16 |
| SPBC24C6.10c | SPCC1827.03c | SPBC17D1.06 | SPAC186.02c | SPBC1773.15 | SPCC4B3.06c |
| SPAC644.08 | SPCC757.12 | SPBC13A2.04c | SPCC550.10 | SPBC1773.17c | SPCC622.21 |
| SPAC1786.02 | SPCC4G3.17 | SPBC4B4.01c | SPBC1348.14c | SPAC57A10.04 | SPAC6C3.03c |
| SPBC4C3.04c | SPCC16C4.08c | SPAC4H3.10c | SPCC338.16 | SPAC3A12.09c | SPCC622.05 |
| SPAC16E8.10c | SPCC4B3.11c | SPAC4H3.12c | SPBC1683.12 | SPCC1682.11c | SPCC584.02 |
| SPBC887.15c | SPCC1322.08 | SPBC1604.09c | SPBC1685.17 | SPBC1685.14c | SPBC17A3.09c |
| SPAC23D3.04c | SPCC584.01c | SPAC22F8.04 | SPAC1A6.01c | SPBC354.12 | SPCC777.10c |
| SPAC1B3.03c | SPAC806.04c | SPBC1198.02 | SPAC20G8.08c | SPAC343.07 | SPCC663.08c |
| SPAPJ691.02 | SPBC106.05c | SPBC1198.07c | SPBC1709.13c | SPBC115.03 | SPCC417.06c |
| SPAC29B12.13 | SPAC227.05 | SPBC839.07 | SPAC23H4.11c | SPBC947.11c | SPCC1450.09c |
| SPAC922.04 | SPBC428.18 | SPAC29B12.10c | SPAC343.21 | SPAC1610.04 | SPBC27.03 |
| SPBC409.08 | SPCC18.12c | SPAC869.10c | SPBP22H7.05c | SPAC1002.01 | SPCC285.07c |
| SPBP22H7.03 | SPBC1685.02c | SPBC27B12.02 | SPBC32H8.13c | SPAPB1A10.08 | SPBC28E12.02 |
| SPAC227.13c | SPAC1296.05c | SPAC630.10 | SPBC28F2.07 | SPCC31H12.06 | SPAC2E1P3.02c |
| SPBC29B5.02c | SPCP1E11.11 | SPAC13C5.05c | SPAC1399.06 | SPAPB1A10.13 | SPAPB24D3.08c |
| SPAC1687.11 | SPBC577.10 | SPAC227.06 | SPBC1A4.03c | SPBC713.06 | SPAPB1A11.01 |
| SPAC1687.16c | SPAC22A12.17c | SPAC227.07c | SPAC17H9.20 | SPBC216.02 | SPCC1620.03 |
| SPBC1E8.05 | SPBC1709.06 | SPBC28F2.09 | SPAC3C7.14c | SPAC607.06c | SPCC1620.12c |
| SPAC22A12.08c | SPBC1306.01c | SPBC1D7.01 | SPBC19G7.01c | SPCC1020.10 | SPCC965.07c |
| SPBC14C8.12 | SPBC21B10.07 | SPAC1687.03c | SPAC1805.03c | SPCC16A11.08 | SPBC16E9.17c |
| SPBC12D12.09 | SPBC16E9.12c | SPBC1711.07 | SPAC24C9.15c | SPBC1709.09 | SPAC688.06c |
| SPBC2G5.03 | SPBP23A10.15c | SPAC10F6.12c | SPAC959.09c | SPBC1709.11c | SPCC569.04 |
| SPAC105.02c | SPAC17H9.19c | SPBC1711.16 | SPAC15A10.16 | SPCC24B10.14c | SPCC569.02c |
| SPAPB1A10.04c | SPAC23C11.08 | SPAC6F12.10c | SPBC1718.02 | SPCC825.04c | SPCC1884.01 |
| SPBP4H10.05c | SPBC15C4.04c | SPBC12C2.04 | SPBC1718.03 | SPBC409.12c | SPCC548.07c |
| SPBP4H10.14c | SPBC24C6.04 | SPBC29A10.08 | SPBC887.16 | SPAC20H4.11c | SPCC594.03 |
| SPAC18G6.12c | SPBC24C6.13 | SPAP27G11.04c | SPBC31F10.04c | SPCC1259.12c | SPCC962.02c |
| SPAC6C3.04 | SPAC15F9.02 | SPBC3E7.10 | SPAC25G10.02 | SPAC13D6.04c | SPCC1183.10 |
| SPBC1105.19 | SPAC19A8.14 | SPBC3E7.11c | SPAC29E6.02 | SPBC651.04 | SPAC323.06c |
| SPAC513.04 | SPAC19A8.05c | SPBC19C7.07c | SPBC1861.01c | SPCC622.02 | SPBC336.07 |
| SPBC244.02c | SPBC29A10.01 | SPBC1703.04 | SPAC12B10.11 | SPAC6C3.05 | SPCPJ732.03 |
| SPBC660.08 | SPAC688.14 | SPAC23C11.09 | SPBC1198.04c | SPCC622.03c | SPAC27E2.08 |
| SPAP7G5.04c | SPBC15D4.03 | SPBC4B4.07c | SPBC660.14 | SPAC6C3.07 | SPCC1020.14 |
| SPAC11G7.01 | SPAC26A3.08 | SPAC17G8.09 | SPBC428.03c | SPCC622.07 | SPCC63.03 |
| SPAPB15E9.01c | SPAC977.12 | SPBC16D10.03 | SPAC19D5.01 | SPCC188.12 | SPCC63.04 |
| SPAC19G12.08 | SPBC776.07 | SPBC31F10.13c | SPAC186.08c | SPCC584.03c | SPBP4H10.09 |
| SPAC2C4.11c | SPAC11D3.04c | SPAC1805.02c | SPBC1709.08 | SPBC32H8.06 | SPCC16A11.06c |
| SPAC16.05c | SPBC19F8.07 | SPAC1805.18 | SPAC212.03 | SPCC777.13 | SPBC2A9.02 |
| SPAC17C9.16c | SPBC11C11.07 | SPBC14F5.05c | SPAC31A2.09c | SPCC417.11c | SPAC25G10.04c |
| SPBC27B12.05 | SPBC13A2.03 | SPAC31G5.09c | SPAC13C5.03 | SPAC19A8.11c | SPCC23B6.03c |
| SPAC1952.04c | SPAC31A2.03 | SPBC1289.07c | SPAPB17E12.03 | SPCC191.04c | SPBC1778.04 |
| SPBC83.13 | SPCC4B3.12 | SPAC2E1P5.05 | SPBC36B7.04 | SPAC23H3.03c | SPCC1281.08 |
| SPAC869.04 | SPBC1677.02 | SPBC31E1.02c | SPBC30D10.10c | SPAC23H3.11c | SPCC622.09 |
| SPAC1F8.07c | SPBC215.01 | SPAP7G5.05 | SPAC18G6.05c | SPAC23H3.13c | SPCC11E10.09c |
| SPBC1711.03 | SPCC1753.04 | SPBC119.15 | SPAC23H3.08c | SPAC23H3.14 | SPCC13B11.04c |
| SPAC3H8.06 | SPBC1652.02 | SPAC458.02c | SPAC1805.04 | SPAC4A8.04 | SPCC777.03c |
| SPAC22A12.06c | SPAC222.12c | SPAC4F10.14c | SPAPB24D3.07c | SPBC2F12.09c | SPBC17D1.07c |
| SPBC30D10.16 | SPCC1223.15c | SPAC5H10.13c | SPBPB10D8.04c | SPAC7D4.11c | SPCC191.09c |
| SPAPB1A10.15 | SPBPB8B6.05c | SPAC13G6.05c | SPBC1198.01 | SPCC18.09c | SPCC191.11 |
| SPAC13G7.04c | SPCPB1C11.02 | SPAC630.08c | SPAC959.05c | SPAPB2B4.04c | SPCC1442.11c |
| SPAC31G5.11 | SPAC10F6.17c | SPAC24H6.10c | SPBC800.12c | SPCC1739.08c | SPCC74.09 |
| SPAC16A10.01 | SPAC6F12.04 | SPBC23G7.09 | SPAC926.02 | SPCPB1C11.01 | SPCC1739.03 |
| SPAC688.03c | SPBC106.01 | SPBC17G9.05 | SPAC14C4.15c | SPAC513.05 | SPAC4F10.08 |
| SPAC1486.09 | SPBC106.17c | SPBP18G5.03 | SPAC977.07c | SPCC126.02c | SPBC31F10.08 |
| SPAC959.11 | SPCC1682.02c | SPBC4F6.09 | SPAC5H10.12c | SPBC3H7.08c | SPAPB8E5.08 |
| SPAC328.09 | SPCC1682.14 | SPBC336.16 | SPAC31A2.08 | SPBP16F5.07 | SPAC1952.15c |
| SPAC16E8.12c | SPAP27G11.03 | SPBC30D10.15 | SPBC15C4.05 | SPBC16E9.16c | SPCC330.19c |
| SPAPB18E9.05c | SPAC343.10 | SPAC1565.04c | SPBC19G7.04 | SPCC1494.09c | SPCC1235.18 |
| SPAC9E9.03 | SPBC839.14c | SPAC20G8.03 | SPAC22G7.07c | SPAC3G9.11c | SPCC794.16 |
| SPAC27D7.03c | SPAC664.04c | SPAPB1A10.03 | SPBC336.05c | SPBC29A3.03c | SPBC1347.03 |
| SPAC1093.02 | SPAC17A5.15c | SPAPB1A10.16 | SPAC9.11 | SPCP1E11.05c | SPCC736.06 |
| SPAC144.01 | SPCC962.04 | SPAC23C11.02c | SPAC23H4.13c | SPCC569.07 | SPBC56F2.03 |
| SPAC17D4.01 | SPAC607.05 | SPAC15E1.08 | SPAC25H1.02 | SPBC18E5.11c | SPBC16A3.13 |
| SPAC1952.03 | SPAC631.01c | SPAC1F7.05 | SPAC7D4.08 | SPBC23G7.13c | SPAC3G6.07 |
| SPAC2H10.01 | SPCC4B3.13 | SPAC27D7.06 | SPAC2E1P3.01 | SPBC21H7.06c | SPBC16C6.14 |
| SPAC11E3.06 | SPCC4B3.07 |  | SPAPB1A11.02 | SPAC6G10.12c | SPCC1259.14c |
| SPAP8A3.10 | SPCC4B3.05c |  | SPAC16A10.05c | SPCC794.03 | SPBPB2B2.07c |
|  | SPCC338.14 |  | SPAC6G9.13c | SPCC794.04c | SPBPB2B2.11 |
|  | SPBC8D2.10c |  | SPAC26A3.09c | SPCC553.05c | SPBPB2B2.12c |
|  | SPCC777.15 |  | SPAC4H3.08 | SPCC736.05 | SPAC869.01 |
|  | SPCC1442.05c |  | SPAC20G4.04c | SPCC736.09c | SPBPB2B2.18 |
|  | SPAC23H3.10 |  | SPAC1039.04 | SPCC736.14 | SPBCPT2R1.04c |
|  | SPAC4A8.07c |  | SPBCPT2R1.07c | SPCC306.10 | SPBCPT2R1.08c |
|  | SPBC2F12.07c |  | SPBC1706.02c | SPCC306.11 | SPAC750.05c |
|  | SPAC644.05c |  | SPAC212.09c | SPCC4G3.12c | SPAC750.07c |
|  | SPCC1739.05 |  | SPCC132.05c | SPCC1672.14 | SPBC1348.07 |
|  | SPCC1739.06c |  | SPAC212.07c | SPBC29A10.02 | SPAC212.12 |
|  | SPBC3H7.13 |  | SPBCPT2R1.10 | SPCC31H12.02c | SPAC212.04c |
|  | SPCC330.07c |  |  | SPAC15A10.05c | SPAC977.18 |
|  | SPAC2E1P5.03 |  |  | SPAP7G5.03 | SPBPB21E7.07 |
|  | SPAC3H5.05c |  |  | SPAP7G5.06 | SPBC359.06 |
|  | SPBC365.14c |  |  | SPBC6B1.02 | SPAC1F8.04c |
|  | SPBC3E7.14 |  |  | SPBC3E7.02c | SPCC622.06c |
|  | SPBC32F12.10 |  |  | SPBC3E7.08c | SPBC1198.12 |
|  | SPBC19C7.05 |  |  | SPCC18B5.05c | SPAC5H10.04 |
|  | SPAC25B8.05 |  |  | SPCC18B5.11c | SPAC5H10.10 |
|  | SPCC1259.05c |  |  | SPBP4H10.10 | SPBC31E1.01c |
|  | SPCC645.04 |  |  | SPBC2A9.07c | SPAC13G6.08 |
|  | SPAC1527.02 |  |  | SPBC15D4.12c | SPBC800.11 |
|  | SPCC777.02 |  |  | SPCC132.03 | SPBC1773.14 |
|  | SPBC3B8.01c |  |  | SPCC338.18 | SPAC1F5.11c |
|  | SPBC2G2.05 |  |  | SPCC338.12 | SPBP26C9.03c |
|  | SPAC20G4.01 |  |  | SPCC338.02 | SPBC1271.06c |
|  | SPBC31F10.06c |  |  | SPBC4C3.12 | SPCC777.04 |
|  | SPCC1840.01c |  |  | SPCC11E10.03 | SPCC663.06c |
|  | SPCC613.02 |  |  | SPAC23D3.16 | SPCC191.10 |
|  | SPCC330.20 |  |  | SPBC609.03 | SPBC428.08c |
|  | SPAC4D7.05 |  |  | SPAC29E6.05c | SPAC13A11.06 |
|  | SPAC869.11 |  |  | SPBC19F8.01c | SPBC1685.06 |
|  | SPAC11D3.06 |  |  | SPCC777.17c | SPAC22F3.04 |
|  | SPCC11E10.01 |  |  | SPAC27D7.04 | SPBC1685.12c |
|  | SPCC777.14 |  |  | SPBC17D1.17 | SPCC1906.04 |
|  | SPAC24H6.11c |  |  | SPCC191.05c | SPCC1620.04c |
|  | SPCC569.08c |  |  | SPAC637.13c | SPCC70.08c |
|  | SPAC1420.03 |  |  | SPAC1093.04c | SPAC23G3.02c |
|  | SPBC4.02c |  |  | SPCC1450.12 | SPBC530.06c |
|  | SPBC4.05 |  |  | SPCC1442.02 | SPBP35G2.06c |
|  | SPAC57A7.12 |  |  | SPBC2G2.15c | SPAC1A6.06c |
|  | SPAC23H4.07c |  |  | SPBC2G2.17c | SPAC5D6.07c |
|  | SPAP27G11.06c |  |  | SPCC1442.17c | SPBC725.06c |
|  | SPBC17A3.04c |  |  | SPBC317.01 | SPBC18H10.09 |
|  | SPAC1399.03 |  |  | SPCC1739.01 | SPBC23G7.10c |
|  | SPBC18H10.13 |  |  | SPAC20G4.02c | SPBC17G9.12c |
|  | SPBC18H10.19 |  |  | SPAC20G4.03c | SPBC14C8.05c |
|  | SPAC3H1.03 |  |  | SPCC576.03c | SPBC14C8.11c |
|  | SPBC12D12.05c |  |  | SPBC23E6.01c | SPAC13F5.03c |
|  | SPAC17G8.08c |  |  | SPCC790.02 | SPBC24C6.09c |
|  | SPAC1B2.04 |  |  | SPCC70.04c | SPBC21D10.08c |
|  | SPAC2E1P3.05c |  |  | SPBC3B9.17 | SPBC21D10.06c |
|  | SPAPB24D3.03 |  |  | SPAC29A4.11 | SPBC6B1.04 |
|  | SPAPB24D3.04c |  |  | SPBC215.08c | SPAC1805.09c |
|  | SPAC31G5.21 |  |  | SPCC330.21 | SPBC685.03 |
|  | SPAC31G5.17c |  |  | SPCC320.06 | SPACUNK4.17 |
|  | SPBC2A9.09 |  |  | SPCC794.01c | SPACUNK4.15 |
|  | SPBC15D4.04 |  |  | SPBC56F2.09c | SPBC2A9.14 |
|  | SPBC15D4.14 |  |  | SPAC14C4.03 | SPAC16A10.08c |
|  | SPAC959.07 |  |  | SPAC14C4.10c | SPAPB1E7.04c |
|  | SPBC19F8.02 |  |  | SPBC56F2.06 | SPAC2E1P5.04c |
|  | SPAC3H5.04 |  |  | SPCC4G3.03 | SPAC26A3.13c |
|  | SPBC25H2.15 |  |  | SPAPJ760.03c | SPBC2G2.10c |
|  | SPAC328.10c |  |  | SPCC10H11.02 | SPAC4H3.05 |
|  | SPBC11C11.09c |  |  | SPBC1861.04c | SPAPB15E9.03c |
|  | SPBC11C11.10 |  |  | SPCP31B10.06 | SPAC27E2.04c |
|  | SPAC1142.02c |  |  | SPCC1672.03c | SPAC19G12.04 |
|  | SPAC15A10.09c |  |  | SPBC1861.05 | SPBC13G1.16 |
|  | SPBC4B4.09 |  |  | SPBC16G5.16 | SPBC215.11c |
|  | SPBC2G2.12 |  |  | SPBC1539.07c | SPBC14F5.12c |
|  | SPBC887.18c |  |  | SPAC1039.05c | SPBC16C6.10 |
|  | SPAC11G7.03 |  |  | SPAC1039.10 | SPAC19B12.12c |
|  | SPBP8B7.21 |  |  | SPAC1039.11c | SPBC1289.17 |
|  | SPBC13G1.01c |  |  | SPBC8E4.01c | SPBPB2B2.13 |
|  | SPBC3B9.22c |  |  | SPCC550.07 | SPBPB2B2.14c |
|  | SPBC215.05 |  |  | SPAC750.02c | SPAC1B3.17 |
|  | SPAC9E9.10c |  |  | SPAC977.01 | SPBPB2B2.19c |
|  | SPAC637.07 |  |  | SPAC977.09c | SPBC1348.02 |
|  | SPAC12B10.06c |  |  | SPBC1683.01 | SPBC1348.04 |
|  | SPAC12B10.09 |  |  | SPAC11D3.02c | SPBPB8B6.02c |
|  | SPBC16G5.01 |  |  | SPCC338.06c | SPBPB21E7.02c |
|  | SPBC16G5.09 |  |  | SPAC11D3.07c | SPBC359.01 |
|  | SPAC20G4.07c |  |  | SPCC622.04 | SPBC359.02 |
|  | SPAPB8E5.02c |  |  | SPAC11D3.14c | SPAC1952.16 |
|  | SPAPB8E5.06c |  |  | SPAC11D3.17 | SPBC800.13 |
|  | SPBPB10D8.06c |  |  | SPAC5H10.01 | SPBC1773.08c |
|  | SPBC660.14 |  |  | SPBC1773.03c | SPAC1006.08 |
|  | SPBC660.16 |  |  | SPBC1773.13 | SPBC1271.08c |
|  | SPAC22E12.14c |  |  | SPBC1773.16c | SPBC428.07 |
|  | SPAC19D5.02c |  |  | SPBP26C9.02c | SPAC13D1.01c |
|  | SPBC839.04 |  |  | SPBC1271.09 | SPBC354.11c |
|  | SPBC530.07c |  |  | SPBC1271.07c | SPBC947.05c |
|  | SPBC646.11 |  |  | SPBC1271.03c | SPBC119.14 |
|  | SPAC3G6.10c |  |  | SPBC1271.01c | SPBC713.07c |
|  | SPBP35G2.09 |  |  | SPBC106.02c | SPAC3G6.01 |
|  | SPBC337.05c |  |  | SPCC417.07c | SPAC29B12.12 |
|  | SPAC806.07 |  |  | SPCC191.06 | SPBC146.11c |
|  | SPBC3D6.08c |  |  | SPCC285.06c | SPAC869.09 |
|  | SPAC630.09c |  |  | SPBC428.06c | SPAC186.03 |
|  | SPBC18H10.02 |  |  | SPCC1223.02 | SPAC750.01 |
|  | SPAC23G3.06 |  |  | SPAC227.15 | SPBC1709.12 |
|  | SPBC3H7.07c |  |  | SPAC2F7.06c | SPAC212.08c |
|  | SPBC1A4.01 |  |  | SPCC18.03 | SPAC977.04 |
|  | SPAC23C4.16c |  |  | SPCC18.04 | SPAC977.06 |
|  | SPAC30D11.14c |  |  | SPAC4G8.09 | SPAPJ695.02 |
|  | SPBC29A3.13 |  |  | SPBC649.05 | SPAC1F8.05 |
|  | SPBC18E5.12c |  |  | SPCC965.04c | SPAC5H10.05c |
|  | SPAC10F6.01c |  |  | SPAC2G11.05c | SPAC12G12.16c |
|  | SPAPB17E12.05 |  |  | SPCC70.07c | SPAC24H6.01c |
|  | SPBC16H5.06 |  |  | SPBC119.04 | SPAC22F3.03c |
|  | SPAC3A12.17c |  |  | SPAC22H12.03 | SPBC1D7.05 |
|  | SPAC9.02c |  |  | SPAC139.04c | SPAC23G3.03 |
|  | SPAC57A7.07c |  |  | SPAC30D11.01c | SPBC18H10.07 |
|  | SPAC343.01c |  |  | SPAC1565.01 | SPBC1E8.04 |
|  | SPAC343.02 |  |  | SPAC3A12.15 | SPAC10F6.15 |
|  | SPBC4F6.11c |  |  | SPBC725.10 | SPAPB17E12.07c |
|  | SPAC3H1.07 |  |  | SPAC57A7.05 | SPAC6F12.09 |
|  | SPBC15D4.05 |  |  | SPAC167.07c | SPBC36B7.06c |
|  | SPBC30D10.08 |  |  | SPAC167.06c | SPAC5D6.09c |
|  | SPBC405.01 |  |  | SPBC8D2.02c | SPBC6B1.03c |
|  | SPAC23C11.05 |  |  | SPBP22H7.09c | SPAC110.02 |
|  | SPBC3B8.08 |  |  | SPBC1215.01 | SPBC21.07c |
|  | SPAC4G9.03 |  |  | SPBC83.12 | SPBC11C11.04c |
|  | SPBC2G2.03c |  |  | SPAC105.03c | SPAC22H10.13 |
|  | SPBC887.13c |  |  | SPAC17A5.18c | SPBC16D10.05 |
|  | SPAC25H1.04 |  |  | SPAC1002.12c | SPAC6B12.16 |
|  | SPBC21C3.08c |  |  | SPBC18H10.05 | SPAC25H1.09 |
|  | SPBC211.08c |  |  | SPAC1399.01c | SPBC16C6.02c |
|  | SPAC644.09 |  |  | SPBC18H10.16 | SPBC1289.14 |
|  | SPBC32C12.03c |  |  | SPAC140.03 | SPBC8E4.05c |
|  | SPAPB2B4.03 |  |  | SPBC16E9.08 | SPBCPT2R1.01c |
|  | SPBC3B9.03 |  |  | SPBC1A4.06c | SPBC1348.03 |
|  | SPBC14F5.09c |  |  | SPBC29A3.14c | SPBC1348.05 |
|  | SPBC16A3.04 |  |  | SPAC3C7.13c | SPBC1348.08c |
|  | SPBC16C6.05 |  |  | SPBC23G7.06c | SPBPB8B6.03 |
|  | SPBC1198.05 |  |  | SPAC20H4.09 | SPBPB8B6.04c |
|  | SPAC959.04c |  |  | SPBC14C8.01c | SPBPB21E7.09 |
|  | SPAC17G6.15c |  |  | SPAC13F5.07c | SPBPB10D8.02c |
|  | SPAC8C9.03 |  |  | SPAC1783.03 | SPAC8E11.08c |
|  | SPAC8C9.09c |  |  | SPAC13D6.01 | SPAC16E8.05c |
|  | SPAC8C9.15c |  |  | SPAC6B12.08 | SPBC1773.05c |
|  | SPBC119.18 |  |  | SPBC12C2.10c | SPBC1773.06c |
|  | SPBC713.03 |  |  | SPBC29A10.15 | SPAC4H3.17 |
|  | SPBC646.03 |  |  | SPAC4F8.08 | SPAC4H3.03c |
|  | SPBC646.05c |  |  | SPBC3E7.06c | SPBC839.06 |
|  | SPAC27D7.07c |  |  | SPAC513.02 | SPAC11G7.06c |
|  | SPAC1834.06c |  |  | SPBC2D10.06 | SPBC119.16c |
|  | SPAC17D4.02 |  |  | SPBC15D4.11c | SPAC1527.01 |
|  | SPAC1782.07 |  |  | SPBC13E7.11 | SPBP35G2.10 |
|  | SPBP22H7.08 |  |  | SPAC6G9.04 | SPAC458.04c |
|  | SPBC83.08 |  |  | SPBC1778.06c | SPBC725.03 |
|  | SPBC27.06c |  |  | SPBC609.04 | SPBC8D2.19 |
|  | SPBC27.08c |  |  | SPBC21.03c | SPAC1006.04c |
|  | SPBC28F2.03 |  |  | SPAP32A8.02 | SPAC29A4.19c |
|  | SPAC13G6.10c |  |  | SPAC328.05 | SPAC14C4.05c |
|  | SPAC18B11.02c |  |  | SPAC15E1.02c | SPAC186.01 |
|  | SPBC3H7.02 |  |  | SPBC2G2.01c | SPAC186.05c |
|  | SPAC630.11 |  |  | SPBP8B7.04 | SPAC186.09 |
|  | SPAC31A2.08 |  |  | SPBP8B7.29 | SPAC750.08c |
|  | SPAC13C5.02 |  |  | SPAC25G10.01 | SPAC212.06c |
|  | SPAC227.12 |  |  | SPAC27F1.05c | SPAC977.14c |
|  | SPBC29A3.11c |  |  | SPAC23D3.17 | SPAC977.16c |
|  | SPBC14C8.07c |  |  | SPBC215.10 | SPAPJ695.01c |
|  | SPBC15C4.01c |  |  | SPAC9E9.02 | SPAC11D3.01c |
|  | SPAC1F3.01 |  |  | SPBC1861.03 | SPAC11D3.09 |
|  | SPBC2G5.06c |  |  | SPBC14F5.04c | SPAC11D3.15 |
|  | SPAC821.08c |  |  | SPAC1093.01 | SPAC5H10.11 |
|  | SPBP4H10.08 |  |  | SPAC11H11.02c | SPBC28E12.06c |
|  | SPAC1A6.10 |  |  | SPBC16C6.06 | SPAC3H8.09c |
|  | SPBC2D10.09 |  |  | SPBC1289.11 | SPAC1D4.14 |
|  | SPBC15D4.06 |  |  | SPBC8E4.04 | SPAC22F3.09c |
|  | SPBC13E7.04 |  |  | SPBPB2B2.01 | SPAC22G7.08 |
|  | SPBC13E7.08c |  |  | SPBPB2B2.02 | SPBC6B1.10 |
|  | SPAC5D6.06c |  |  | SPAC1B3.06c | SPAC1A6.11 |
|  | SPAC824.04 |  |  | SPAC1B3.20 | SPAC23H4.05c |
|  | SPAP11E10.01 |  |  | SPBPB2B2.17c | SPAC1610.03c |
|  | SPAC9G1.05 |  |  | SPBC359.05 | SPAC1002.16c |
|  | SPAC3C7.02c |  |  | SPAC1952.17c | SPAC9G1.09 |
|  | SPAC3C7.03c |  |  | SPBC1683.08 | SPAC17H9.01 |
|  | SPAC4G9.11c |  |  | SPBC1683.13c | SPAC13F5.06c |
|  | SPAC17G8.07 |  |  | SPBC660.05 | SPAC17G8.14c |
|  | SPAC823.15 |  |  | SPBC1773.12 | SPAC15F9.01c |
|  | SPAC644.18c |  |  | SPAC26F1.11 | SPAC6B12.06c |
|  | SPAC1805.06c |  |  | SPAPJ691.02 | SPAC23H3.15c |
|  | SPAC1805.07c |  |  | SPAPJ760.02c | SPAC4A8.05c |
|  | SPACUNK4.06c |  |  | SPBC354.13 | SPACUNK4.20 |
|  | SPAC24C9.03 |  |  | SPAP8A3.04c | SPAC513.06c |
|  | SPAC589.09 |  |  | SPBC947.06c | SPAC2E1P3.03c |
|  | SPAC3G9.03 |  |  | SPBC577.13 | SPAPB1A11.03 |
|  | SPAC1486.11 |  |  | SPBC36.13 | SPAC589.02c |
|  | SPAC959.08 |  |  | SPBP35G2.17 | SPAC3G9.12 |
|  | SPAC3A11.07 |  |  | SPAC1039.09 | SPAC6G10.06 |
|  | SPAC1556.05c |  |  | SPAC922.07c | SPAC26A3.02 |
|  | SPAC4H3.07c |  |  | SPAC5H10.02c | SPAC8E11.07c |
|  | SPAC25G10.05c |  |  | SPAC5H10.06c | SPAC3H5.09c |
|  | SPAC30C2.05 |  |  | SPBC725.12 | SPAC3A11.03 |
|  | SPAC144.11 |  |  | SPAC1F5.09c | SPAC17A2.11 |
|  | SPAC22F8.02c |  |  | SPBC3D6.11c | SPAPB15E9.06 |
|  | SPAC1B3.01c |  |  | SPAC227.14 | SPAC27F1.10 |
|  | SPAC29A4.20 |  |  | SPBC29B5.01 | SPAC27D7.12c |
|  | SPAP8A3.06 |  |  | SPAC22F3.13 | SPAC4F10.17 |
|  | SPAC29B12.05c |  |  | SPBC2F12.05c | SPAC1952.01 |
|  |  |  |  | SPBC18H10.10c | SPAC1250.07 |
|  |  |  |  | SPAC222.04c | SPAC1250.02 |
|  |  |  |  | SPAC139.03 | SPAC869.06c |
|  |  |  |  | SPAC56F8.06c | SPAC186.04c |
|  |  |  |  | SPBC29A3.17 | SPAC186.06 |
|  |  |  |  | SPAC10F6.07c | SPAC186.07c |
|  |  |  |  | SPBC1711.11 | SPBCPT2R1.06c |
|  |  |  |  | SPBC1711.12 | SPCC548.02c |
|  |  |  |  | SPBC16H5.14c | SPCC18B5.02c |
|  |  |  |  | SPAC1565.07c | SPCP20C8.03 |
|  |  |  |  | SPAC23H4.01c | SPBC1348.11 |
|  |  |  |  | SPBC3E7.04c | SPAC977.13c |
|  |  |  |  | SPBC3E7.09 | SPCC576.16c |
|  |  |  |  | SPAC824.05 | SPBC18E5.15 |
|  |  |  |  | SPAC17A5.11 | SPBPB10D8.03 |
|  |  |  |  | SPAC1399.05c | SPBC31A8.02 |
|  |  |  |  | SPBC685.05 | SPBPB21E7.06 |
|  |  |  |  | SPBC13E7.09 | SPAC23D3.05c |
|  |  |  |  | SPAC607.09c |  |
|  |  |  |  | SPBC776.16 |  |
|  |  |  |  | SPBC21.02 |  |
|  |  |  |  | SPBC19F8.06c |  |
|  |  |  |  | SPBC17D11.03c |  |
|  |  |  |  | SPBC2G2.04c |  |
|  |  |  |  | SPAC6C3.04 |  |
|  |  |  |  | SPBC887.06c |  |
|  |  |  |  | SPAC6B12.03c |  |
|  |  |  |  | SPBP8B7.27 |  |
|  |  |  |  | SPBP8B7.30c |  |
|  |  |  |  | SPBC25D12.02c |  |
|  |  |  |  | SPAC23H3.04 |  |
|  |  |  |  | SPBC21C3.20c |  |
|  |  |  |  | SPBC1604.01 |  |
|  |  |  |  | SPAPB2B4.06 |  |
|  |  |  |  | SPAC6F6.02c |  |
|  |  |  |  | SPBC215.07c |  |
|  |  |  |  | SPBC1347.07 |  |
|  |  |  |  | SPBC56F2.15 |  |
|  |  |  |  | SPBC1861.06c |  |
|  |  |  |  | SPBC14F5.10c |  |
|  |  |  |  | SPAC8F11.05c |  |
|  |  |  |  | SPACUNK4.12c |  |
|  |  |  |  | SPBC16G5.15c |  |
|  |  |  |  | SPBC16A3.02c |  |
|  |  |  |  | SPAC589.11 |  |
|  |  |  |  | SPAC688.08 |  |
|  |  |  |  | SPBC1539.02 |  |
|  |  |  |  | SPAC3G9.13c |  |
|  |  |  |  | SPBC1289.16c |  |
|  |  |  |  | SPBP4G3.02 |  |
|  |  |  |  | SPBPB2B2.06c |  |
|  |  |  |  | SPBPB2B2.09c |  |
|  |  |  |  | SPBPB2B2.10c |  |
|  |  |  |  | SPAC6G10.03c |  |
|  |  |  |  | SPAC6G10.08 |  |
|  |  |  |  | SPBPB21E7.04c |  |
|  |  |  |  | SPAPB1E7.08c |  |
|  |  |  |  | SPBC1683.04 |  |
|  |  |  |  | SPAC3A11.05c |  |
|  |  |  |  | SPAC17A2.02c |  |
|  |  |  |  | SPBC1271.14 |  |
|  |  |  |  | SPBC1271.05c |  |
|  |  |  |  | SPBC1271.04c |  |
|  |  |  |  | SPBC106.03 |  |
|  |  |  |  | SPBC106.11c |  |
|  |  |  |  | SPBC1685.11 |  |
|  |  |  |  | SPAC1556.06 |  |
|  |  |  |  | SPBC354.15 |  |
|  |  |  |  | SPAC323.07c |  |
|  |  |  |  | SPBC839.08c |  |
|  |  |  |  | SPAC2F3.08 |  |
|  |  |  |  | SPAPB18E9.02c |  |
|  |  |  |  | SPAC27E2.07 |  |
|  |  |  |  | SPAC26H5.09c |  |
|  |  |  |  | SPAC25B8.08 |  |
|  |  |  |  | SPAC2C4.17c |  |
|  |  |  |  | SPAC23D3.11 |  |
|  |  |  |  | SPBC216.05 |  |
|  |  |  |  | SPAC9E9.11 |  |
|  |  |  |  | SPAC1851.04c |  |
|  |  |  |  | SPAC27D7.09c |  |
|  |  |  |  | SPAC637.03 |  |
|  |  |  |  | SPAC1093.07 |  |
|  |  |  |  | SPBC409.11 |  |
|  |  |  |  | SPAC19B12.08 |  |
|  |  |  |  | SPBC27B12.08 |  |
|  |  |  |  | SPBC27B12.14 |  |
|  |  |  |  | SPAC1B3.10c |  |
|  |  |  |  | SPAC29A4.12c |  |
|  |  |  |  | SPAC26F1.02 |  |
|  |  |  |  | SPAC11E3.05 |  |
|  |  |  |  | SPAC3G6.05 |  |
|  |  |  |  | SPAC1039.06 |  |
|  |  |  |  | SPAC1039.08 |  |
|  |  |  |  | SPAC922.05c |  |
|  |  |  |  | SPAC869.08 |  |
|  |  |  |  | SPAC977.10 |  |
|  |  |  |  | SPAC11D3.19 |  |
|  |  |  |  | SPAC11D3.11c |  |
|  |  |  |  | SPBC21B10.09 |  |
|  |  |  |  | SPBC18H10.08c |  |
|  |  |  |  | SPAC12G12.12 |  |
|  |  |  |  | SPBC16H5.13 |  |
|  |  |  |  | SPBC12D12.02c |  |
|  |  |  |  | SPAC1F3.03 |  |
|  |  |  |  | SPAC1F3.10c |  |
|  |  |  |  | SPBC557.05 |  |
|  |  |  |  | SPAC1296.03c |  |
|  |  |  |  | SPBC29A10.14 |  |
|  |  |  |  | SPAC4G8.04 |  |
|  |  |  |  | SPBC685.02 |  |
|  |  |  |  | SPAC222.15 |  |
|  |  |  |  | SPAC821.04c |  |
|  |  |  |  | SPAC139.05 |  |
|  |  |  |  | SPBC32F12.17 |  |
|  |  |  |  | SPBC2D10.04 |  |
|  |  |  |  | SPBC2D10.14c |  |
|  |  |  |  | SPAC10F6.13c |  |
|  |  |  |  | SPAPB17E12.09 |  |
|  |  |  |  | SPAP27G11.08c |  |
|  |  |  |  | SPAC343.13 |  |
|  |  |  |  | SPAC1002.10c |  |
|  |  |  |  | SPAC607.08c |  |
|  |  |  |  | SPAC23C11.15 |  |
|  |  |  |  | SPAC13F5.04c |  |
|  |  |  |  | SPAC4G9.07 |  |
|  |  |  |  | SPAC13G7.02c |  |
|  |  |  |  | SPAC13G7.07 |  |
|  |  |  |  | SPAC19A8.16 |  |
|  |  |  |  | SPAC4A8.02c |  |
|  |  |  |  | SPAC823.16c |  |
|  |  |  |  | SPAC644.13c |  |
|  |  |  |  | SPAC3F10.07c |  |
|  |  |  |  | SPAC3F10.13 |  |
|  |  |  |  | SPACUNK4.19 |  |
|  |  |  |  | SPAPB24D3.10c |  |
|  |  |  |  | SPAC31G5.12c |  |
|  |  |  |  | SPAC1786.01c |  |
|  |  |  |  | SPAC589.07c |  |
|  |  |  |  | SPAC3A11.11c |  |
|  |  |  |  | SPAC3A11.06 |  |
|  |  |  |  | SPAC328.08c |  |
|  |  |  |  | SPAC1556.01c |  |
|  |  |  |  | SPAC4H3.04c |  |
|  |  |  |  | SPAC1071.11 |  |
|  |  |  |  | SPAC2F3.15 |  |
|  |  |  |  | SPAC25B8.02 |  |
|  |  |  |  | SPAC683.03 |  |
|  |  |  |  | SPAC1F7.06 |  |
|  |  |  |  | SPAC1F7.09c |  |
|  |  |  |  | SPAC29E6.07 |  |
|  |  |  |  | SPAC27D7.03c |  |
|  |  |  |  | SPAC27D7.11c |  |
|  |  |  |  | SPAC27D7.13c |  |
|  |  |  |  | SPAC17D4.01 |  |
|  |  |  |  | SPAC11H11.01 |  |
|  |  |  |  | SPAC20G4.05c |  |
|  |  |  |  | SPAC4F10.07c |  |
|  |  |  |  | SPAC1952.13 |  |
|  |  |  |  | SPAC2E12.03c |  |
|  |  |  |  | SPAC26F1.01 |  |
|  |  |  |  | SPAC4D7.02c |  |
|  |  |  |  | SPAC29B12.14c |  |
|  |  |  |  | SPAC1039.01 |  |
|  |  |  |  | SPAC869.07c |  |
|  |  |  |  | SPAC869.03c |  |
|  |  |  |  | SPAC750.03c |  |
|  |  |  |  | SPBPB21E7.08 |  |
|  |  |  |  | SPCC830.02 |  |
