# Supplementary material for: The longevity and reversibility of quiescence in Schizosaccharomyces pombe are dependent upon the HIRA histone chaperone
Source: Cell Cycle. 2023 Aug 27;22(17):1921–36. doi: 10.1080/15384101.2023.2249705 (PMC10599175; doi:10.1080/15384101.2023.2249705)
Supplement: Supplemental Material [file KCCY_A_2249705_SM9609.zip › Table S3.docx]

**Table S3. Biological process GO terms associated with genes differentially expressed in *hip1*Δ**

**(A) Decreased in G0**

No significant enrichment

**(B) Decreased in Exit**

| External ID | GO term | Over represented / Under represented | Corrected  p value |
| --- | --- | --- | --- |
| GO:0009123 | nucleoside monophosphate metabolic process | Over represented | 0.000608997 |
| GO:0009144 | purine nucleoside triphosphate metabolic process | Over represented | 0.000608997 |
| GO:0044237 | cellular metabolic process | Over represented | 0.000608997 |
| GO:0006412 | translation | Over represented | 0.000949034 |
| GO:0009205 | purine ribonucleoside triphosphate metabolic process | Over represented | 0.000949034 |
| GO:0008152 | metabolic process | Over represented | 0.0010195 |
| GO:0002181 | cytoplasmic translation | Over represented | 0.00151638 |
| GO:0009126 | purine nucleoside monophosphate metabolic process | Over represented | 0.00151638 |
| GO:0009141 | nucleoside triphosphate metabolic process | Over represented | 0.00151638 |
| GO:0009161 | ribonucleoside monophosphate metabolic process | Over represented | 0.00151638 |
| GO:0009167 | purine ribonucleoside monophosphate metabolic process | Over represented | 0.00151638 |
| GO:0009199 | ribonucleoside triphosphate metabolic process | Over represented | 0.00151638 |
| GO:0044281 | small molecule metabolic process | Over represented | 0.00151638 |
| GO:0006163 | purine nucleotide metabolic process | Over represented | 0.00152375 |
| GO:0009150 | purine ribonucleotide metabolic process | Over represented | 0.00155593 |
| GO:0009259 | ribonucleotide metabolic process | Over represented | 0.00157959 |
| GO:0044249 | cellular biosynthetic process | Over represented | 0.00157959 |
| GO:0071704 | organic substance metabolic process | Over represented | 0.00179033 |
| GO:0009260 | ribonucleotide biosynthetic process | Over represented | 0.00221373 |
| GO:0009152 | purine ribonucleotide biosynthetic process | Over represented | 0.00236051 |
| GO:0044238 | primary metabolic process | Over represented | 0.00253619 |
| GO:1901564 | organonitrogen compound metabolic process | Over represented | 0.00283151 |
| GO:0009058 | biosynthetic process | Over represented | 0.00290075 |
| GO:0009124 | nucleoside monophosphate biosynthetic process | Over represented | 0.00290075 |
| GO:0046034 | ATP metabolic process | Over represented | 0.00290075 |
| GO:0046390 | ribose phosphate biosynthetic process | Over represented | 0.00290075 |
| GO:1901576 | organic substance biosynthetic process | Over represented | 0.00290075 |
| GO:0009987 | cellular process | Over represented | 0.00346042 |
| GO:0044711 | single-organism biosynthetic process | Over represented | 0.00375786 |
| GO:0019693 | ribose phosphate metabolic process | Over represented | 0.00429346 |
| GO:0009127 | purine nucleoside monophosphate biosynthetic process | Over represented | 0.00511594 |
| GO:0009168 | purine ribonucleoside monophosphate biosynthetic process | Over represented | 0.00511594 |
| GO:0006164 | purine nucleotide biosynthetic process | Over represented | 0.00563934 |
| GO:0009156 | ribonucleoside monophosphate biosynthetic process | Over represented | 0.00563934 |
| GO:0042455 | ribonucleoside biosynthetic process | Over represented | 0.00829214 |
| GO:0006520 | cellular amino acid metabolic process | Over represented | 0.00969226 |

**(C) Decreased in G0 and Exit**

| External ID | GO term | Over represented / Under represented | Corrected  p value |
| --- | --- | --- | --- |
| GO:0019236 | response to pheromone | Over represented | 6.50E-05 |
| GO:0071444 | cellular response to pheromone | Over represented | 6.50E-05 |
| GO:0000750 | pheromone-dependent signal transduction involved in conjugation with cellular fusion | Over represented | 0.000192301 |
| GO:0032005 | signal transduction involved in conjugation with cellular fusion | Over represented | 0.000319812 |
| GO:0007186 | G-protein coupled receptor signaling pathway | Over represented | 0.0009766 |
| GO:0000749 | response to pheromone involved in conjugation with cellular fusion | Over represented | 0.00209143 |
| GO:0007166 | cell surface receptor signaling pathway | Over represented | 0.00209143 |
| GO:0000746 | conjugation | Over represented | 0.008522 |
| GO:0000747 | conjugation with cellular fusion | Over represented | 0.008522 |

**(D) Increased in G0**

No significant enrichment

**(E) Increased in Exit**

| External ID | GO term | Over represented / Under represented | Corrected  p value |
| --- | --- | --- | --- |
| GO:0010467 | gene expression | Under represented | 1.47E-09 |
| GO:0042254 | ribosome biogenesis | Under represented | 1.47E-09 |
| GO:0043170 | macromolecule metabolic process | Under represented | 1.47E-09 |
| GO:0044260 | cellular macromolecule metabolic process | Under represented | 2.88E-09 |
| GO:0044085 | cellular component biogenesis | Under represented | 4.71E-09 |
| GO:0022613 | ribonucleoprotein complex biogenesis | Under represented | 9.32E-09 |
| GO:0071840 | cellular component organization or biogenesis | Under represented | 2.72E-08 |
| GO:0034470 | ncRNA processing | Under represented | 3.18E-07 |
| GO:0006412 | translation | Under represented | 1.22E-06 |
| GO:0009059 | macromolecule biosynthetic process | Under represented | 1.31E-06 |
| GO:0034645 | cellular macromolecule biosynthetic process | Under represented | 1.46E-06 |
| GO:0044238 | primary metabolic process | Under represented | 1.46E-06 |
| GO:0002181 | cytoplasmic translation | Under represented | 1.48E-06 |
| GO:0034660 | ncRNA metabolic process | Under represented | 1.48E-06 |
| GO:0044237 | cellular metabolic process | Under represented | 1.48E-06 |
| GO:0044249 | cellular biosynthetic process | Under represented | 1.91E-06 |
| GO:0009058 | biosynthetic process | Under represented | 2.26E-06 |
| GO:0008152 | metabolic process | Under represented | 2.51E-06 |
| GO:0009987 | cellular process | Under represented | 2.51E-06 |
| GO:0071704 | organic substance metabolic process | Under represented | 2.51E-06 |
| GO:1901576 | organic substance biosynthetic process | Under represented | 5.16E-06 |
| GO:0016072 | rRNA metabolic process | Under represented | 1.77E-05 |
| GO:0006364 | rRNA processing | Under represented | 2.53E-05 |
| GO:0044267 | cellular protein metabolic process | Under represented | 0.000102682 |
| GO:0019538 | protein metabolic process | Under represented | 0.00011412 |
| GO:0006396 | RNA processing | Under represented | 0.000123676 |
| GO:0016070 | RNA metabolic process | Under represented | 0.000364531 |
| GO:0090304 | nucleic acid metabolic process | Under represented | 0.00125952 |
| GO:0006139 | nucleobase-containing compound metabolic process | Under represented | 0.00217929 |
| GO:0065003 | macromolecular complex assembly | Under represented | 0.00255817 |
| GO:0016043 | cellular component organization | Under represented | 0.00381032 |
| GO:0006865 | amino acid transport | Over represented | 0.00477227 |
| GO:0046483 | heterocycle metabolic process | Under represented | 0.00608391 |
| GO:1901360 | organic cyclic compound metabolic process | Under represented | 0.00676693 |
| GO:0006725 | cellular aromatic compound metabolic process | Under represented | 0.00864893 |
| GO:0006820 | anion transport | Over represented | 0.0090604 |

**(F) Increased in G0 and Exit**

| External ID | GO term | Over represented /  Under represented | Corrected  p value |
| --- | --- | --- | --- |
| GO:0009987 | cellular process | Under represented | 8.79E-16 |
| GO:0008152 | metabolic process | Under represented | 6.69E-15 |
| GO:0044238 | primary metabolic process | Under represented | 1.33E-14 |
| GO:0044237 | cellular metabolic process | Under represented | 1.85E-14 |
| GO:0071704 | organic substance metabolic process | Under represented | 1.85E-14 |
| GO:0010467 | gene expression | Under represented | 2.93E-14 |
| GO:0009058 | biosynthetic process | Under represented | 2.82E-11 |
| GO:0043170 | macromolecule metabolic process | Under represented | 6.02E-11 |
| GO:0044249 | cellular biosynthetic process | Under represented | 9.44E-11 |
| GO:1901576 | organic substance biosynthetic process | Under represented | 1.47E-10 |
| GO:0044260 | cellular macromolecule metabolic process | Under represented | 1.54E-10 |
| GO:0016070 | RNA metabolic process | Under represented | 1.48E-08 |
| GO:0019538 | protein metabolic process | Under represented | 3.20E-07 |
| GO:1901360 | organic cyclic compound metabolic process | Under represented | 5.91E-07 |
| GO:0044267 | cellular protein metabolic process | Under represented | 7.77E-07 |
| GO:0006807 | nitrogen compound metabolic process | Under represented | 1.34E-06 |
| GO:0007126 | meiotic nuclear division | Over represented | 1.34E-06 |
| GO:0030437 | ascospore formation | Over represented | 1.34E-06 |
| GO:0048468 | cell development | Over represented | 1.34E-06 |
| GO:0030435 | sporulation resulting in formation of a cellular spore | Over represented | 1.81E-06 |
| GO:0034293 | sexual sporulation | Over represented | 1.81E-06 |
| GO:0043934 | sporulation | Over represented | 1.81E-06 |
| GO:0043935 | sexual sporulation resulting in formation of a cellular spore | Over represented | 1.81E-06 |
| GO:0006725 | cellular aromatic compound metabolic process | Under represented | 2.22E-06 |
| GO:0048646 | anatomical structure formation involved in morphogenesis | Over represented | 2.22E-06 |
| GO:0046483 | heterocycle metabolic process | Under represented | 2.36E-06 |
| GO:0015074 | DNA integration | Over represented | 5.65E-06 |
| GO:0006412 | translation | Under represented | 8.42E-06 |
| GO:0006139 | nucleobase-containing compound metabolic process | Under represented | 1.06E-05 |
| GO:0034641 | cellular nitrogen compound metabolic process | Under represented | 1.48E-05 |
| GO:0051321 | meiotic cell cycle | Over represented | 1.64E-05 |
| GO:0009059 | macromolecule biosynthetic process | Under represented | 2.43E-05 |
| GO:0044085 | cellular component biogenesis | Under represented | 2.43E-05 |
| GO:0034645 | cellular macromolecule biosynthetic process | Under represented | 3.15E-05 |
| GO:0022413 | reproductive process in single-celled organism | Over represented | 4.49E-05 |
| GO:0032505 | reproduction of a single-celled organism | Over represented | 4.49E-05 |
| GO:0034306 | regulation of sexual sporulation | Over represented | 5.19E-05 |
| GO:0034307 | regulation of ascospore formation | Over represented | 5.19E-05 |
| GO:0042173 | regulation of sporulation resulting in formation of a cellular spore | Over represented | 5.19E-05 |
| GO:0043937 | regulation of sporulation | Over represented | 5.19E-05 |
| GO:0043940 | regulation of sexual sporulation resulting in formation of a cellular spore | Over represented | 5.19E-05 |
| GO:0060284 | regulation of cell development | Over represented | 5.19E-05 |
| GO:1903046 | meiotic cell cycle process | Over represented | 5.91E-05 |
| GO:0090304 | nucleic acid metabolic process | Under represented | 6.92E-05 |
| GO:0006396 | RNA processing | Under represented | 8.76E-05 |
| GO:1901362 | organic cyclic compound biosynthetic process | Under represented | 9.72E-05 |
| GO:0030154 | cell differentiation | Over represented | 9.93E-05 |
| GO:0034470 | ncRNA processing | Under represented | 0.000111015 |
| GO:0071840 | cellular component organization or biogenesis | Under represented | 0.000117456 |
| GO:0044711 | single-organism biosynthetic process | Under represented | 0.000123052 |
| GO:0003006 | developmental process involved in reproduction | Over represented | 0.00014817 |
| GO:0010721 | negative regulation of cell development | Over represented | 0.000174747 |
| GO:0042174 | negative regulation of sporulation resulting in formation of a cellular spore | Over represented | 0.000174747 |
| GO:0043939 | negative regulation of sporulation | Over represented | 0.000174747 |
| GO:0043942 | negative regulation of sexual sporulation resulting in formation of a cellular spore | Over represented | 0.000174747 |
| GO:0044702 | single organism reproductive process | Over represented | 0.000174747 |
| GO:0045596 | negative regulation of cell differentiation | Over represented | 0.000174747 |
| GO:0051093 | negative regulation of developmental process | Over represented | 0.000174747 |
| GO:0075297 | negative regulation of ascospore formation | Over represented | 0.000174747 |
| GO:0034660 | ncRNA metabolic process | Under represented | 0.000380401 |
| GO:0043933 | macromolecular complex subunit organization | Under represented | 0.000380908 |
| GO:0045595 | regulation of cell differentiation | Over represented | 0.000452383 |
| GO:0018130 | heterocycle biosynthetic process | Under represented | 0.000459991 |
| GO:0008150 | biological process | Under represented | 0.000582177 |
| GO:0019438 | aromatic compound biosynthetic process | Under represented | 0.000603742 |
| GO:0051641 | cellular localization | Under represented | 0.00109026 |
| GO:0044265 | cellular macromolecule catabolic process | Under represented | 0.0011695 |
| GO:0034622 | cellular macromolecular complex assembly | Under represented | 0.00163596 |
| GO:0045835 | negative regulation of meiotic nuclear division | Over represented | 0.00165887 |
| GO:0034654 | nucleobase-containing compound biosynthetic process | Under represented | 0.00168431 |
| GO:0000280 | nuclear division | Over represented | 0.00196542 |
| GO:0019222 | regulation of metabolic process | Under represented | 0.00201677 |
| GO:0045184 | establishment of protein localization | Under represented | 0.0021197 |
| GO:0033036 | macromolecule localization | Under represented | 0.00281089 |
| GO:0044699 | single-organism process | Under represented | 0.00281089 |
| GO:0046907 | intracellular transport | Under represented | 0.00281089 |
| GO:0080090 | regulation of primary metabolic process | Under represented | 0.00284774 |
| GO:0031323 | regulation of cellular metabolic process | Under represented | 0.00322061 |
| GO:0048285 | organelle fission | Over represented | 0.003256 |
| GO:0065003 | macromolecular complex assembly | Under represented | 0.00351477 |
| GO:0009057 | macromolecule catabolic process | Under represented | 0.00376913 |
| GO:0015031 | protein transport | Under represented | 0.00376913 |
| GO:0044710 | single-organism metabolic process | Under represented | 0.00426391 |
| GO:0044271 | cellular nitrogen compound biosynthetic process | Under represented | 0.00507088 |
| GO:0060255 | regulation of macromolecule metabolic process | Under represented | 0.00507088 |
| GO:0048518 | positive regulation of biological process | Under represented | 0.00558866 |
| GO:0000003 | reproduction | Over represented | 0.00614068 |
| GO:1902582 | single-organism intracellular transport | Under represented | 0.00683305 |
| GO:0008104 | protein localization | Under represented | 0.00695006 |
| GO:0040020 | regulation of meiotic nuclear division | Over represented | 0.00786323 |
| GO:0051649 | establishment of localization in cell | Under represented | 0.00865075 |
| GO:0006886 | intracellular protein transport | Under represented | 0.00886711 |
| GO:0070727 | cellular macromolecule localization | Under represented | 0.00886711 |
