# Supplementary material for: The longevity and reversibility of quiescence in Schizosaccharomyces pombe are dependent upon the HIRA histone chaperone
Source: Cell Cycle. 2023 Aug 27;22(17):1921–36. doi: 10.1080/15384101.2023.2249705 (PMC10599175; doi:10.1080/15384101.2023.2249705)
Supplement: Supplemental Material [file KCCY_A_2249705_SM9609.zip › Table S4.docx]

**Table S4. Nucleoside metabolic process genes that are differentially regulated (< -1 Log2 FC) in *hip1*Δ during exit from G0.**

| Gene name | Ensembl Gene ID | Description |
| --- | --- | --- |
|  | SPBC1198.05 | guanylate kinase (predicted) [Source:PomBase;Acc:SPBC1198.05] |
|  | SPBC660.16 | phosphogluconate dehydrogenase, decarboxylating [Source:PomBase;Acc:SPBC660.16] |
| *tim11* | SPBC106.05C | F0-ATPase subunit E (predicted) [Source:PomBase;Acc:SPBC106.05c] |
| *hdd1* | SPCC4G3.17 | HD domain protein (predicted) [Source:PomBase;Acc:SPCC4G3.17] |
| *cox4* | SPAC1296.02 | cytochrome c oxidase subunit IV (predicted) [Source:PomBase;Acc:SPAC1296.02] |
| *atp2* | SPAC222.12C | F1-ATPase beta subunit Atp2 [Source:PomBase;Acc:SPAC222.12c] |
| *cox9* | SPCC1259.05C | cytochrome c oxidase subunit VIIa (predicted) [Source:PomBase;Acc:SPCC1259.05c] |
| *ura5* | SPBC725.15 | orotate phosphoribosyltransferase Ura5 [Source:PomBase;Acc:SPBC725.15] |
| *ado1* | SPCC338.14 | adenosine kinase (predicted) [Source:PomBase;Acc:SPCC338.14] |
| *adk1* | SPAC4G9.03 | adenylate kinase Adk1 [Source:PomBase;Acc:SPAC4G9.03] |
| *rip1* | SPBC16H5.06 | ubiquinol-cytochrome-c reductase complex subunit 5 [Source:PomBase;Acc:SPBC16H5.06] |
| *ade5* | SPCC569.08C | phosphoribosylglycinamide formyltransferase (predicted) [Source:PomBase;Acc:SPCC569.08c] |
| *dut1* | SPAC644.05C | deoxyuridine 5'-triphosphate nucleotidohydrolase (predicted) [Source:PomBase;Acc:SPAC644.05c] |
| *cox6* | SPAC1B2.04 | cytochrome c oxidase subunit VI (predicted) [Source:PomBase;Acc:SPAC1B2.04] |
| *qcr10* | SPBP4H10.08 | Reiske ISP-associated protein, ubiquinol-cytochrome-c reductase complex subunit Qcr10 (predicted) [Source:PomBase;Acc:SPBP4H10.08] |
| *atp16* | SPBC13E7.04 | F1-ATPase delta subunit (predicted) [Source:PomBase;Acc:SPBC13E7.04] |
| *ade1* | SPBC405.01 | phosphoribosylamine-glycine ligase/phosphoribosylformylglycinamidine cyclo-ligase [Source:PomBase;Acc:SPBC405.01] |
| *nde2* | SPAC3A11.07 | mitochondrial NADH dehydrogenase (ubiquinone) Nde2 (predicted) [Source:PomBase;Acc:SPAC3A11.07] |
| *atp4* | SPBC1604.07 | F0-ATPase subunit (predicted) [Source:PomBase;Acc:SPBC1604.07] |
| *ade8* | SPBC14F5.09C | adenylosuccinate lyase Ade8 [Source:PomBase;Acc:SPBC14F5.09c] |
| *qcr8* | SPAC1782.07 | ubiquinol-cytochrome-c reductase complex subunit 7 [Source:PomBase;Acc:SPAC1782.07] |
|  | SPAC1B3.01C | uracil phosphoribosyltransferase (predicted) [Source:PomBase;Acc:SPAC1B3.01c] |
