# Supplementary material for: The longevity and reversibility of quiescence in Schizosaccharomyces pombe are dependent upon the HIRA histone chaperone
Source: Cell Cycle. 2023 Aug 27;22(17):1921–36. doi: 10.1080/15384101.2023.2249705 (PMC10599175; doi:10.1080/15384101.2023.2249705)
Supplement: Supplemental Material [file KCCY_A_2249705_SM9609.zip › Table S5.docx]

**Table S5. Impact of HIRA on the expression of MBF subunit genes**

| **MBF subunit** | **Log2 fold change in mRNA *wt* vs *hip1*Δ** | |
| --- | --- | --- |
|  | **G0** | **Exit** |
| *res1^+^* (SPBC725.16) | -0.872 | -0.589 |
| *res2^+^* SPAC22F3.09c | 1.081 | 1.183 |
| *cdc10^+^* (SPBC336.12c) | -0.015 | -0.244 |
| *rep2^+^* (SPBC2F12.11c) | 0.430 | -1.731 |
| *yox1^+^* (SPBC21B10.13c) | 0.071 | -0.983 |
| *nrm1^+^* (SPBC16A3.07c) | 0.204 | 0.704 |
